# Flame-retardant Cl-substituted electrolyte for low-temperature and high-voltage lithium-ion batteries with fast interfacial kinetics

**DOI:** 10.1093/nsr/nwaf420

**Published:** 2025-09-27

**Authors:** Yujie Yang, Jinyu Zhang, Huaqing Yu, Xu Liu, Yifei Liu, Boyuan Li, Jia Li, Shuangxin Ren, Zhenyu Fan, Yawen Li, Kun Li, Lanqing Wu, Qifang Gao, Zhenhua Yan, Xin Gao, Qing Zhao

**Affiliations:** State Key Laboratory of Advanced Chemical Power Sources, Frontiers Science Center for New Organic Matter, Key Laboratory of Advanced Energy Materials Chemistry (Ministry of Education), College of Chemistry, Nankai University, Tianjin 300071, China; School of Materials Science and Engineering, Peking University, Beijing 100871, China; Center for Nanoscale Science and Technology, Academy for Advanced Interdisciplinary Studies, Peking University, Beijing 100871, China; State Key Laboratory of Advanced Chemical Power Sources, Frontiers Science Center for New Organic Matter, Key Laboratory of Advanced Energy Materials Chemistry (Ministry of Education), College of Chemistry, Nankai University, Tianjin 300071, China; State Key Laboratory of Advanced Chemical Power Sources, Frontiers Science Center for New Organic Matter, Key Laboratory of Advanced Energy Materials Chemistry (Ministry of Education), College of Chemistry, Nankai University, Tianjin 300071, China; State Key Laboratory of Advanced Chemical Power Sources, Frontiers Science Center for New Organic Matter, Key Laboratory of Advanced Energy Materials Chemistry (Ministry of Education), College of Chemistry, Nankai University, Tianjin 300071, China; State Key Laboratory of Advanced Chemical Power Sources, Frontiers Science Center for New Organic Matter, Key Laboratory of Advanced Energy Materials Chemistry (Ministry of Education), College of Chemistry, Nankai University, Tianjin 300071, China; State Key Laboratory of Advanced Chemical Power Sources, Frontiers Science Center for New Organic Matter, Key Laboratory of Advanced Energy Materials Chemistry (Ministry of Education), College of Chemistry, Nankai University, Tianjin 300071, China; State Key Laboratory of Advanced Chemical Power Sources, Frontiers Science Center for New Organic Matter, Key Laboratory of Advanced Energy Materials Chemistry (Ministry of Education), College of Chemistry, Nankai University, Tianjin 300071, China; State Key Laboratory of Advanced Chemical Power Sources, Frontiers Science Center for New Organic Matter, Key Laboratory of Advanced Energy Materials Chemistry (Ministry of Education), College of Chemistry, Nankai University, Tianjin 300071, China; State Key Laboratory of Advanced Chemical Power Sources, Frontiers Science Center for New Organic Matter, Key Laboratory of Advanced Energy Materials Chemistry (Ministry of Education), College of Chemistry, Nankai University, Tianjin 300071, China; State Key Laboratory of Advanced Chemical Power Sources, Frontiers Science Center for New Organic Matter, Key Laboratory of Advanced Energy Materials Chemistry (Ministry of Education), College of Chemistry, Nankai University, Tianjin 300071, China; State Key Laboratory of Advanced Chemical Power Sources, Frontiers Science Center for New Organic Matter, Key Laboratory of Advanced Energy Materials Chemistry (Ministry of Education), College of Chemistry, Nankai University, Tianjin 300071, China; State Key Laboratory of Advanced Chemical Power Sources, Frontiers Science Center for New Organic Matter, Key Laboratory of Advanced Energy Materials Chemistry (Ministry of Education), College of Chemistry, Nankai University, Tianjin 300071, China; State Key Laboratory of Advanced Chemical Power Sources, Frontiers Science Center for New Organic Matter, Key Laboratory of Advanced Energy Materials Chemistry (Ministry of Education), College of Chemistry, Nankai University, Tianjin 300071, China; State Key Laboratory of Advanced Chemical Power Sources, Frontiers Science Center for New Organic Matter, Key Laboratory of Advanced Energy Materials Chemistry (Ministry of Education), College of Chemistry, Nankai University, Tianjin 300071, China; School of Materials Science and Engineering, Peking University, Beijing 100871, China; Center for Nanoscale Science and Technology, Academy for Advanced Interdisciplinary Studies, Peking University, Beijing 100871, China; State Key Laboratory of Advanced Chemical Power Sources, Frontiers Science Center for New Organic Matter, Key Laboratory of Advanced Energy Materials Chemistry (Ministry of Education), College of Chemistry, Nankai University, Tianjin 300071, China

**Keywords:** Li-ion batteries, Cl-based electrolyte, flame retardant, high-voltage, low-temperature

## Abstract

Carbonate electrolytes such as ethylene carbonate and dimethyl carbonate are excellent at stabilizing the graphite (Gr) anode and thus enable the unprecedented success of lithium-ion batteries but suffer from high flammability and slow ion-transport kinetics at low temperature. Here, we propose a chlorine atom substitution strategy to address the long-standing challenge of carbonate-based electrolytes. Cl atom substitution with an electron-withdrawing effect can facilitate the interfacial reaction by weakening the interactions with Li^+^ and forming a solid electrolyte interphase involving LiCl, as well as terminating the chain reaction of combustion when releasing Cl radicals. At a conventional salt concentration (1 M), the Cl-substituted carbonate electrolyte achieves stable operation of the LiNi_0.8_Co_0.1_Mn_0.1_O_2_ (NCM811) cathode at a high cut-off voltage (4.6 V) and low-temperature adaptation towards the Gr anode (91.9% capacity retention at −20°C). The Ah-level Gr/NCM811 pouch cell maintains 84.6% capacity retention over 300 cycles and can enable the rigorous nail penetration short-circuit test at a fully charged state. This work provides a promising approach to build cost-efficient electrolytes for safe and energy-dense lithium-ion batteries with wide-temperature application potentials.

## Teaser

This study reports a chlorine-substituted carbonate electrolyte for 4.6-V lithium-ion batteries, in which the released Cl^•^ radicals act as flame retardants to increase safety, the weakened Li+-solvent coordination and a LiCl-involved SEI accelerate the interfacial reaction at low-temperature, and the anion-rich solvation structrue enables the formation of the robost CEI to suppress aluminum corrosion with enhanced high-voltage stability.

## INTRODUCTION

As electrical energy storage/conversion devices, lithium-ion batteries (LIBs) have been widely used in various portable devices and electric vehicle power systems [1–4]. Graphite anodes currently dominate the LIB market due to their low Li-ion intercalation potential (<0.1 V vs Li/Li^+^) and excellent cycling stability [5,6]. In particular, combining a high voltage cathode such as Ni-rich LiNi_0.8_Co_0.1_Mn_0.1_O_2_ (NCM811), the energy density of LIBs can be further enhanced by elevating the charging cut-off voltage [1,7–12]. The electrolyte is known to play an important role in the electrochemical performance of LIBs, because it is not only responsible for the ion transport in the battery, but also determines the composition of the solid electrolyte interface (SEI) and the desolvation process of Li ions [13–15]. At present, ethylene carbonate (EC) is still an essential component of commercial electrolytes for its capability of forming a stable SEI on the graphite surface, and dissociating Li salts at the very highest level, but its high melting point and high viscosity limit its application under extreme conditions [16–18]. Although electrolytes with faster ion motion and lower viscosity were obtained by mixing linear carbonates such as dimethyl carbonate (DMC), the strong affinity between Li^+^ and EC fails to provide a fast desolvation process and increases the risk of lithium dendrite plating under low-temperature or fast-charging conditions [5,17,19]. To make matters worse, the carbonate-based electrolytes are generally known to be highly flammable, imposing severe safety risks for applications [20,21].

To fulfill the requirements of LIBs working under extreme conditions, various types of electrolytes have been developed in recent years. Electrolytes based on ether solvents are considered to be a potential substitute for carbonate solvents due to their low viscosity and low melting point, especially for some ether molecules with weak solvation ability [for example, tetrahydrofuran (THF) and 1,2-diethoxyethane (DEE)] that have demonstrated superior fast charging and low temperature performance [22–26]. However, the poor oxidation stability (<4.0 V vs Li^+^/Li) of ether molecules is incompatible with high-voltage cathodes and thus lose the possibility to build energy-dense LIBs [27–29]. Furthermore, ethers are highly flammable with extremely low flashpoints (for example, −21°C for THF) [30]. Introducing fluorinated fragments can effectively improve the oxidation stability of ether electrolytes [31,32], while the strong electron absorption of fluorine will sacrifice the capability of ion dissociation, especially at the increased F atom number that is considered to exhibit better flame-retardant ability [30,33,34]. Alternatively, phosphonate solvents such as trimethyl phosphate and triethyl phosphate have also been known to be flame-retardant solvents [35–38], but their high binding energy towards Li^+^ leads to solvent co-intercalation into the graphite layer with a subsequent decomposition reaction [33]. Building high-concentration electrolytes using Li salts such as lithium bis(fluorosulfonyl)imide (LiFSI) can achieve solvent-free intercalation through the construction of an inorganic-rich SEI derived from an anion-rich Li-ion solvation structure [39,40]. However, the high viscosity and low conductivity make it less possible for low-temperature or fast-charging application [41]. Therefore, there is an urgent need to develop electrolyte solvent molecules that are endowed with flame retardancy, oxidation stability and rapid electrochemical reaction kinetics.

Similar to a fluorine atom, a chlorine atom also demonstrates a certain electron withdrawing ability, and thus the introduction of a Cl atom can also lower the highest occupied molecular orbital (HOMO) energy level of the molecule [42]. Simultaneously, the electron withdrawing ability of Cl atoms can decrease the electron cloud density of O atoms, thereby reducing the coordination ability with Li^+^ and accelerating the desolvation process. In addition, the combustion mechanism of electrolytes is mainly attributed to the chain reaction caused by the generation of active H^•^ radicals in organic solvents [43]. The binding energy of C–Cl is generally lower than that of C–F, making it easier to release Cl radicals to capture the H radicals (Cl^•^ + H^•^ → HCl) and further capture the HO^•^ radicals (HCl + HO^•^ → Cl^•^ + H_2_O) to terminate the chain reaction of combustion [44]. Finally, the reduction of Cl-substituted solvent at lower potential is supposed to generate an SEI rich in LiCl, which in principle exhibits a lower Li-ion diffusion energy barrier than LiF [45,46]. Therefore, through rational design, the electrolytes using Cl-substituted solvent are promising to build high-safety and energy-dense LIBs that can enable fast-charging and low-temperature conditions.

Herein, using DMC as the basic electrolyte solvent, we study how Cl substitution regulates the electrochemical performance of LIBs. The combustion test first reveals the rapid flame-retardant effect of chloromethyl methyl carbonate (CMMC) solvent. When using as an electrolyte solvent, the combination of CMMC with fluoroethylene carbonate (5% FEC) enables long-term co-intercalation-free stability towards the graphite (Gr) anode. Meanwhile, the introduction of a Cl atom is not only beneficial for the Li-ion desolvation process due to its electron withdrawing ability, but also increases Li-ion transport through the interphase due to the formation of an SEI involving LiCl. Furthermore, the weak coordination between CMMC and Li^+^ increases the participation of anions (FSI^−^) in the solvation structure, suppressing the corrosion of the Al current collector by FSI^−^ and largely enhancing the oxidation stability of the electrolyte. Consequently, the designed electrolyte at a conventional concentration (1 M LiFSI) enables stable cycling of the Gr/NCM811 full cell under high voltage (4.6 V), as well as excellent high-rate (5 C) and low-temperature performance (−20°C). The Ah-level Gr/NCM811 pouch cell can maintain over 800 cycles with a capacity retention of 61.18% (84.55% after 300 cycles). Cl substitution provides a feasible approach for designing electrolytes with high-safety, high-voltage stability and fast ion desolvation and transport process through the electrolyte/electrode interphase.

## RESULTS AND DISCUSSION

### Design principle of Cl-substituted carbonate electrolyte

**Scheme 1.**
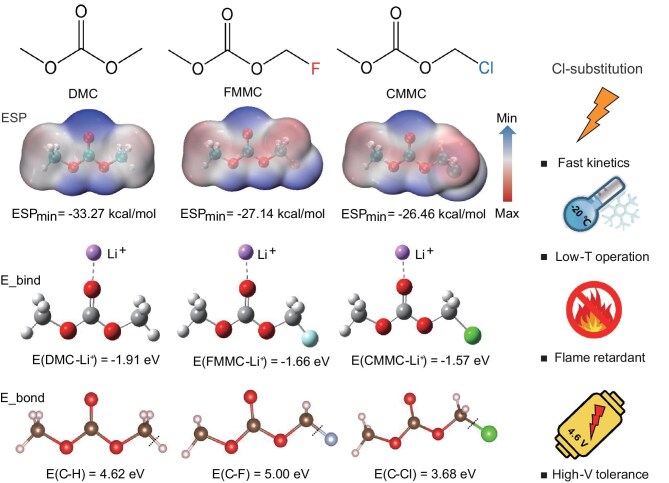
Merits of Cl-substituted carbonate electrolyte. Among the studied molecules, the ESP_min_ of Cl substitution was the highest, thus revealing the most positive binding energy to Li^+^. In addition, the lowest bond energy of C–Cl is beneficial for the formation of Cl^•^ to terminate the chain reaction of combustion. As a result, the CMMC-based electrolyte is endowed with fast kinetics, low-temperature operation, flame retardancy and high-voltage tolerance.

The design principle of Cl-substituted carbonate electrolytes is described in Scheme 1. DMC was chosen for molecular design because of its low viscosity relative to most carbonate molecules, contributing to fast ion transport dynamics in the bulk electrolyte [17]. We also compare F and Cl substitution on DMC through theoretical calculation. Electrostatic potential (ESP) mapping was applied to unveil the most negative site of the molecule, which prefers to coordinate with Li^+^. It is found that the carbonyl is endowed with minimal ESP and the value is the most positive in the CMMC molecule, indicating the weakest coordination capability to Li^+^. This phenomenon is also confirmed by the value of the binding energy with Li^+^. In addition, the binding energy of C–H, C–F and C–Cl was also calculated to study the feasibility of chemical bond breaking. The lowest binding energy of C–Cl indicates that the formation of Cl^•^ is the easiest, thus providing excellent flame-retardant capability. As confirmed by an ignition experiment, the DMC is easily ignited and continuously burns with a blue flame (Fig. S1a, Video S1). In sharp contrast, the CMMC solvent is not burning until the second ignition process and immediately self-extinguishing (Fig. S1b, Video S2), which proves the excellent flame-retardant effect of the Cl functional group. In short, due to the high electronegativity, the introduction of a Cl atom can reduce the electron densities of oxygen atoms, resulting in weak coordination between the O atom and Li^+^ and further accelerating the desolvation process of Li^+^. In addition, the reduction of CMMC is supposed to generate a LiCl-rich SEI, facilitating the transport of Li through the interphase.

**Figure 1.**
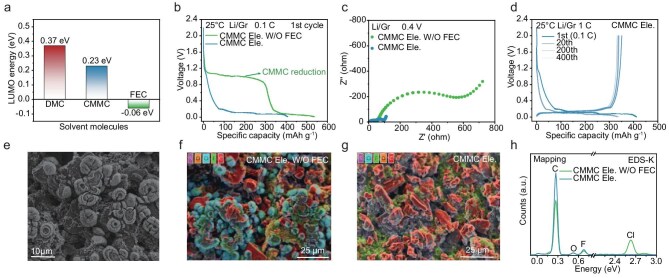
Increasing compatibility of CMMC electrolytes with graphite anodes. (a) LUMO energy levels of DMC, CMMC and FEC molecules. (b) Initial galvanostatic discharge profiles and (c) corresponding EIS spectra at 0.4 V for Li/Gr half-cells using CMMC Ele. or CMMC Ele. without (W/O) FEC (0.1 C). (d) Charge/discharge profiles of Li/Gr half-cells using CMMC Ele. within 400 cycles (1 C). (e) SEM morphology of graphite electrodes discharged to 0.8 V using CMMC Ele. W/O FEC. Elemental mapping (EDS) of graphite surfaces using (f) CMMC Ele. W/O FEC and (g) CMMC Ele. (h) Comparative EDS spectra of graphite surfaces with the above two electrolytes. The concentration of electrolyte was 1 M LiFSI.

To verify our hypothesis, we first studied the electrochemical performance of a CMMC electrolyte with 1 M LiFSI as the Li salt with a Gr anode, which usually lays the foundation for the application of LIBs (Table S1). Unfortunately, due to the reduced lowest unoccupied molecular orbital (LUMO) energy of CMMC (Fig. 1a, Fig. S2, Table S2), there is a long reduction peak at around 1.1 V before Li-ion intercalation during the initial galvanostatic discharge process (Fig. 1b), leading to an extremely low initial Coulombic efficiency (CE) of 36.45% and rapid battery failure (Fig. S3). Through comparing the LUMO energy, FEC was added to the electrolyte as a sacrificial agent, in order to prevent the excessive reduction of CMMC as the LUMO energy of FEC is lower than that of CMMC under both free and coordinated states (Fig. 1a and Fig. S2). We found that a 5% FEC additive is optimum considering both stability and cost (Fig. S4), in which the Li/Gr half-cell shows a typical Li^+^ intercalation reaction with a high initial CE of 85.29% (Fig. S5). Simultaneously, the electrochemical impedance spectroscopy (EIS) spectra also reveal the largely reduced interfacial resistance after adding FEC (Fig. 1c). A long-cycling test using an electrolyte of 1 M LiFSI in CMMC with 5% FEC demonstrates that the capacity retention of the Gr anode is 98.5% after 400 cycles at 1 C (1 C = 372 mA g^−1^) with average CE over 99.9% (Fig. 1d and Fig. S5).

To unveil the composition of the reducing substances generated at 1.1 V and the function of FEC, we first used scanning electron microscopy (SEM) to observe the surface of graphite in Li/Gr half-cells after discharging to 0.8 V. As shown in Fig. 1e, there are a large number of circular by-products attached to graphite grains. Combining with energy dispersive spectroscopy (EDS) analysis, it can be found that the distribution position of Cl is consistent with the shape of by-products (Fig. 1f, Figs S6 and S7), indicating the Cl-rich composition. Upon adding 5% FEC, the surface of the Gr anode is almost intact (Fig. 1g and Fig. S8) with largely weakened Cl signal (Fig. 1h and Fig. S9). X-ray diffraction (XRD) patterns of the Gr anode after discharging to 0.8 V provided more direct information about the by-product, in which typical LiCl peaks located at 2θ = 30.2° and 35.0° have been found without FEC addition (Fig. S10). The excessive decomposition of CMMC leads to the formation of thick SEI and fast consumption of electrolytes, further causing premature failure of batteries. When the FEC additive is added to a 1 M LiFSI/CMMC electrolyte, the C–Cl stretching vibration peak of CMMC is nearly unchanged (Fig. S11), indicating that FEC doesn’t enhance the C–Cl bond through molecular interactions. Therefore, FEC is suggested to form a protection film on the Gr anode, suppressing the over-reduction of CMMC and resulting in excellent durability of Li||Gr electrochemical cells. Consequently, 1 M LiFSI + 5% FEC in CMMC electrolyte (CMMC Ele.) was selected for further research.

### Solvation structures and ion transport mechanism of electrolytes

**Figure 2.**
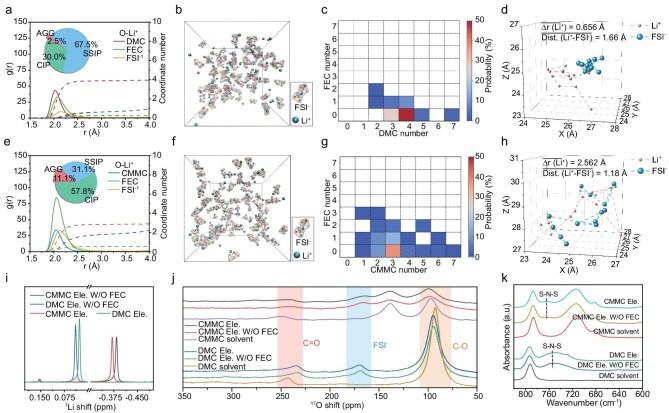
Solvation structures and ion transport mechanisms. (a–h) Molecular dynamic simulations of (a–d) DMC Ele. and (e–h) CMMC Ele. (a and e) Radial distribution function (RDF) with CN of Li^+^; (b and f) snapshots of MD simulation; (c and g) dynamic composition of solvation structure; (d and h) Li^+^ and FSI^−^ movement trajectory at continuous frames. (i–k) Spectral characterization: (i) ^7^Li, (j) ^17^O NMR spectra and (k) ATR-FTIR spectra of studied electrolytes and solvents.

As the aforementioned study suggests that the coordination between Li^+^ and solvent in CMMC electrolyte is weaker than that in DMC, we then further used molecular dynamics (MD) simulation to investigate the solvation structure of Li ions, in which 1 M LiFSI + 5% FEC in DMC electrolyte (DMC Ele.) is also compared as the control group (Fig. 2a–h, Tables S3 and S4). The average coordination number (CN) for each Li^+^ is 3.91 for the O in DMC and 0.70 for the O in FSI^−^ in DMC-based electrolyte (Fig. 2a), while the value in CMMC-based electrolyte is 2.86 and 1.88 for the O in CMMC and FSI^−^, respectively (Fig. 2e). This result confirms the weakened solvation ability of CMMC, resulting in less coordination between Li ions and solvents and more coordination between Li ions and anions. Meanwhile, the FEC also participates in the solvation structure with a coordination ratio of 71% (Fig. S12), which ensures the participation of FEC in the formation of the SEI and suppression of the excess decomposition of CMMC.

The Li^+^ solvation structures were further classified according to the participation of anions in coordination, named as solvent-separated ion pairs [SSIPs, (Li^+^)_1_(FSI^−^)_0_], contact ion pairs [CIPs, (Li^+^)_1_(FSI^−^)_1_] and ion aggregates [AGGs, (Li^+^)_≥2_(FSI^−^)_1_]. In comparison with DMC electrolyte, which is rich in SSIP solvation structure (67.5%), the anion-participating coordination is more common for CMMC electrolytes (57.8% for CIP and 11.1% for AGG) (Fig. 2a, b, e and f). In order to unveil the potential effects of FEC additives, the dynamic specific composition of the solvation structure was analyzed through the solvent molecule confusion matrix (Fig. 2c and g). It can be found that compared with DMC electrolyte, FEC molecules in CMMC electrolyte are more prone to enter the first solvation structure, ensuring the formation of a stable SEI. In addition, the trajectories of Li ions and FSI ions during MD simulation were analyzed (Fig. 2d and h). The average displacement of Li ions in CMMC electrolyte (2.562 Å) is much longer than that in DMC electrolyte (0.656 Å), indicating the low ion migration barrier. The phenomenon also suggests that CMMC electrolyte is endowed with a greater ligand exchange Li ion migration mechanism than in DMC electrolyte, as confirmed by the higher consistency between the movement trajectories of Li^+^ and FSI^−^ with shortened average distance (1.18 vs 1.66 Å).

We then provided more detailed information on the solvation structure with spectra approaches. Nuclear magnetic resonance (NMR) can be used to detect the chemical environment of the element. The magnetic field generated by the flowing electrons around the atom leads to a chemical shift. As shown in Fig. 2i, the ^7^Li peak of the two CMMC electrolytes shows a significant negative chemical shift compared with those of DMC electrolytes, indicating a higher electron cloud density around Li^+^. Combining the above MD simulation results, we suppose that the higher electron density around Li^+^ in a CMMC electrolyte is due to the increased CN for FSI^−^. The electron density of the O atoms on the solvent will increase after coordinating with Li^+^ due to the ion–dipole interaction, resulting in a negative chemical shift of the ^17^O peak [47]. As shown in Fig. 2j, the ^17^O peaks of the C=O and C–O in CMMC (or DMC) solvent appear as a negative and positive chemical shift, respectively, indicating that the solvent mainly coordinates with Li^+^ through the O in C=O. Compared with DMC electrolyte, the ^17^O peak of FSI^−^ in CMMC electrolyte shows a more negative chemical shift, illustrating that the FSI^−^ in CMMC electrolyte has a stronger ion–ion interaction with Li^+^ due to the weak solvation ability of CMMC. We also investigated the interactions between Li^+^ and FSI^−^ by attenuated total reflectance Fourier transform infrared (ATR-FTIR) spectroscopy. The S–N–S vibration peak of FSI^−^ of DMC electrolyte shows a significant red shift compared to CMMC electrolyte, indicating that there are more free anions in DMC electrolyte [48], owing to the stronger solvation ability of DMC, which provides better dissociation ability for LiFSI (Fig. 2k and Fig. S13). Furthermore, Raman spectra also revealed a higher proportion of AGGs in the CMMC electrolyte compared to the DMC electrolyte (Fig. S14).

In conclusion, the above results confirm the weak solvation ability of CMMC, resulting in a anion-rich solvation structure. As the negligible influence of the interaction between Li^+^ and anions on the desolvation process, the weaker binding between CMMC with Li^+^ will promote the electrochemical reaction kinetics [49]. The anion-rich solvation structure will also contribute to an anion-derived robust electrode/electrolyte interphase [25,50], which is beneficial for ion conduction and enhances the mechanical properties of SEI/cathode electrolyte interphase (CEI).

### Electrochemical performance of LIBs

**Figure 3.**
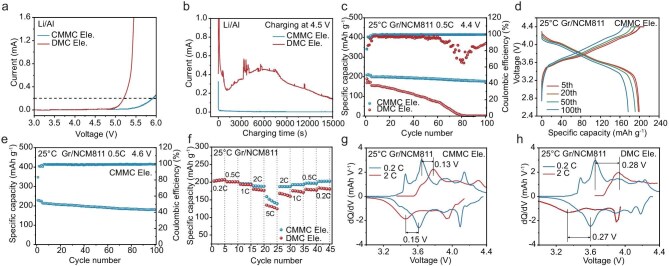
Electrochemical performance of high-voltage LIBs at room temperature. (a) LSV (1 mV s^−1^) and (b) 4.5 V CV floating test in Li/Al cells for oxidation stability evaluation. (c) Specific capacity and CE of the Gr/NMC811 full cells at 4.4 V. (d) The corresponding galvanostatic discharge/charge profiles of CMMC Ele. at different cycles. (e) Specific capacity and CE of the Gr/NMC811 full cells at 4.6 V. The above cells were charged/discharged at 0.1 C (2 cycles) and 0.2 C (2 cycles) for formation cycles and then cycled at 0.5 C plus CC/CV charging procedure with a cut-off current of 0.1 C. (f) Rate performance of the two electrolytes. The corresponding d*Q*/d*V* curves of the (g) CMMC Ele. and (h) DMC Ele. at different current rates. The cells for rate performance test were evaluated using the CC/CV charging procedure with a cut-off current of 0.1 C. The average mass loading of the cathode was 7.85 mg cm^−2^ and the capacity ratio of the anode to the cathode was ∼1.06. Both DMC and CMMC Ele. were tested for comparison.

To evaluate the stability of CMMC-based electrolyte under high voltage, we first assembled Li/Al cells for linear sweep voltammetry (LSV) testing. As shown in Fig. 3a, the decomposition potential of the CMMC electrolyte is about 0.7 V higher than that of the DMC electrolyte. Correspondingly, a significant corrosion current continues to occur in the DMC electrolyte during the 4.5 V constant-voltage polarization test, whereas the corrosion current for the CMMC electrolyte was almost negligible (Fig. 3b). These results indicate the improved oxidation stability achieved by chlorine functionality, which can be caused by the reduced Al corrosion due to fewer free FSI^−^ ions in the CMMC electrolyte [51,52]. Constant-voltage float charging tests from 4.3 to 4.6 V in Li/NCM811 cells also confirmed the exceptional oxidation stability of the CMMC electrolyte (Fig. S15). We then studied the electrochemical properties of the two electrolytes with NCM811 cathodes matching Gr anodes. Figure 3c, d and Fig. S16 show the cycle performance of Gr/NCM811 cells for the two electrolytes at 0.5 C and 4.4 V cut-off voltage (1 C = 188 mA g^−1^). The average CE within 100 cycles is 99.4% for the CMMC electrolyte with a capacity retention of 88.7%. However, the specific capacity for the DMC electrolyte continues to decrease with almost no capacity remaining after 100 cycles.

In order to distinguish the effects of different electrolytes on the cathode and anode, respectively, we assembled Li/NCM811 and Li/Gr half-cells for further investigation. The Li/Gr half-cell with the DMC electrolyte doesn’t show obvious capacity decrease within 400 cycles (Fig. S17), while the Li/NCM811 cell shows strong capacity decrease (Figs S18 and S19), which is similar to the behavior in the Gr/NCM811 full cell. This result indicates that the incompatibility between DMC electrolyte and NCM811 cathode at 4.4 V is the main reason for its poor electrochemical performance. More intriguingly, the Gr/NCM811 full cell with the CMMC electrolyte can achieve a higher average specific capacity of 196.1 mAh g^−1^ at 0.5 C and an extremely high cut-off voltage of 4.6 V, and the capacity retention is still 84.4% after 100 cycles with an average CE of 99.2% (Fig. 3e). In comparison, the batteries with the DMC electrolyte even struggle to sustain five cycles (Fig. S20). The long constant voltage (CV) charging process of Gr/NCM811 cell with the DMC electrolyte indicates the serious Al current collector corrosion caused by FSI^−^.

We also evaluated the feasibility of the CMMC electrolyte for LIBs under fast-charging conditions. As shown in Fig. 3f, the CMMC electrolyte retains a discharge specific capacity of 159.5 mAh g^−1^ at 5 C, and reveals good reversibility after returning to the low current density. Through comparing with d*Q*/d*V* differential and discharge/charge curves (Fig. 3g and h, Fig. S21), we find that the electrochemical reaction peaks of the DMC electrolyte shift more significantly with increasing current, and the polarization voltage of the DMC electrolyte at 5 C is about 0.4 V higher than that of the CMMC electrolyte. These results illustrate that the CMMC electrolyte has faster electrochemical reaction kinetics, which benefits from both weak solvation ability and interfacial superiority of CMMC electrolyte. The Li^+^ transference number of the CMMC electrolyte is 0.595, which is higher than that of the DMC electrolyte (0.478) (Fig. S22). Moreover, the CMMC electrolyte is also beneficial for lithium metal striping/plating (Fig. S23), further mitigating capacity loss caused by irreversible lithium deposition during fast-charging processes. The cycle performance of the Gr/NCM811 full cell was further evaluated for a 1 C charging and 0.2 C discharging process, which is more in line with the actual usage conditions (Fig. S24). The average specific capacity of the CMMC electrolyte was 193.0 mAh g^−1^ and the average CE was 99.6% within 100 cycles. In comparison, the DMC electrolyte showed a significant capacity decrease from the 50th cycle and only 23.6 mAh g^−1^ specific capacity remained after 100 cycles. To sum up, CMMC electrolyte is a feasible solution for fast-charging and high-voltage LIBs.

### Low-temperature superiority of LIBs using CMMC electrolyte

**Figure 4.**
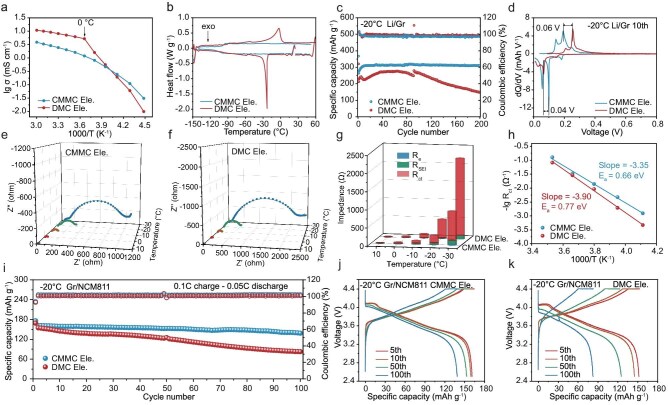
Low-temperature performance and charge-transfer kinetics of LIBs. (a) Ionic conductivity versus temperature of CMMC and DMC Ele. (b) DSC endothermic/exothermic curves of the above electrolytes. (c) Cycling performance of Li/Gr (0.6 mAh cm^−2^) half-cells at −20°C. (d) The corresponding d*Q*/d*V* curves of the CMMC Ele. for the 10th cycle. EIS spectra of (e) CMMC Ele. and (f) DMC Ele. at different temperatures. (g) Fitted values of *R*_SEI_, *R*_ct_ and *R*_e_ of the above electrolytes. (h) The activation energy of the charge transfer process obtained by Arrhenius fitting. (i) Cycling performance of Gr/NCM811 full cells at −20°C. The corresponding galvanostatic profiles of (j) CMMC Ele. and (k) DMC Ele. at different cycles. The above cells were charged/discharged at 0.2 C for 5 formation cycles at room temperature at full lithiation state of the Gr anode before transferring to −20°C. The average mass loading of NCM811 was 7.85 mg cm^−2^ and the capacity ratio of the anode to the cathode was ∼1.06.

Another advantage of chlorination is the excellent electrochemical performance at low temperature. As shown in Fig. 4a, Fig. S25 and Table S5, although the DMC electrolytes have higher ionic conductivity at relatively high temperature, they show a sharp decrease in conductivity near 0°C, indicating the solidification of electrolyte and the precipitation of lithium salt at this temperature [7]. In comparison, the conductivity of the CMMC electrolytes doesn’t show a sharp decrease with temperature and surpass DMC electrolytes near −30°C (CMMC: 0.25 mS cm^−1^ vs DMC: 0.19 mS cm^−1^). Differential scanning calorimetry (DSC) measurement demonstrates that only the DMC electrolyte exhibits a phase transition peak at −23°C (Fig. 4b), which is consistent with the sharp decrease in conductivity of the DMC electrolytes mentioned above.

The low melting point of CMMC electrolytes triggers our interest to evaluate the electrochemical performance of Gr anodes at low temperature. As shown in Fig. 4c, d and Fig. S26, the discharge specific capacity of the Li/Gr half-cell with the CMMC electrolyte can retain a high specific capacity above 310 mAh g^−1^ within 200 cycles at −20°C, corresponding to a 91.9% capacity retention relative to room temperature. In comparison, the specific capacity of the DMC electrolyte decreased to 148.1 mAh g^−1^ after the 200th cycle. The dQ/dV differential curves at the 10th cycle show that there are three peaks during both the discharge and charge processes, corresponding to the three stages of Li-ion intercalation/de-intercalation into the graphite layer (Fig. 4d). Among them, the first two strongest peaks of the DMC electrolyte are 0.06 and 0.04 V higher than that of the CMMC electrolyte, respectively, confirming the fast intercalation kinetics of the CMMC electrolyte at low temperature.

As the electrochemical reaction kinetics mainly depend on the electrolyte ionic conductivity, desolvation energy and SEI resistance [53–55], we then analyzed the impact of these three factors on the reaction kinetics through EIS at different temperatures. To eliminate the interference of Li metal, the Gr anode was removed from a fully discharged Li/Gr half-cell and a Gr/Gr symmetric cell was reassembled with the same electrolyte to conduct the EIS measurement. The equivalent circuit was employed to fit the Nyquist plots and to obtain the resistance of the bulk electrolyte (*R*_e_), SEI (*R*_SEI_) and charge transfer process (*R*_ct_) (Fig. 4e and f and Fig. S27). As the results summarized in Fig. 4g show, the *R*_ct_ increases most rapidly and becomes significantly larger than both *R*_e_ and *R*_SEI_, particularly at low temperatures, indicating that the rate-determining step of the electrochemical reactions at low temperature is the charge transfer process. We then fitted the *R*_ct_ using the Arrhenius equation $\frac{1}{R} = \frac{1}{{{R}_0}}{e}^{ - {E}_{\mathrm{a}}/{K}_{\mathrm{B}}T}$ to obtain the activation energy (*E*_a_) [56], which was 0.66 and 0.77 eV for CMMC and DMC electrolytes, respectively (Fig. 4h). As the charge transfer process is mainly dominated by the Li^+^-desolvation process [57], we can conclude that the weak solvation ability of CMMC promotes the electrochemical reaction kinetics at low temperature. As a result, the Gr/NCM811 full cell using CMMC electrolyte at −20°C achieves an average specific capacity of 152.3 mAh g^−1^ within 100 cycles, largely exceeding that of the DMC electrolyte (82.4 mAh g^−1^) (Fig. 4i–k). In summary, a chlorine substitution strategy proposes a successful solution for fast reaction kinetics at low temperatures.

### Electrode/electrolyte interphase at low/high voltage

**Figure 5.**
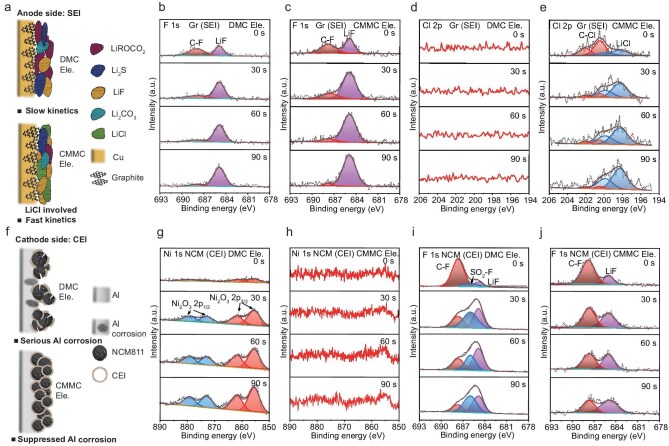
Composition of electrode/electrolyte interphase characterized by XPS. (a–e) SEI on the Gr anode and (f–j) CEI on the NCM811 cathode. (a) Schematic diagram of SEI, showing that SEI formed by CMMC Ele. is endowed with faster kinetics as it is rich in LiCl and other inorganic compounds. (b and c) F 1s and (d and e) Cl 2p spectra of the SEI. (f) Schematic diagram of CEI, showing that CEI formed by CMMC Ele. is intact and can suppress the Al corrosion of electrolyte. (g and h) Ni 1s and (i and j) F 1s spectra of the CEI on the NCM811 cathode. The electrolytes are (b, d, g, i) DMC Ele. and (c, e, h, j) CMMC Ele., respectively. All elements were calibrated according to the reference value of 284.6 eV for adsorbed carbon.

A stable electrode/electrolyte interphase with fast ion conductivity and good protective ability is also critical for the electrochemical performance of batteries. In order to analyze the chemical composition of the SEI/CEI on the graphite anode or the NCM811 cathode, the cycled electrodes (5 cycles) were characterized by X-ray photoelectron spectroscopy (XPS), respectively. On the Gr anode, stronger LiF and Li_2_S signals were found for the CMMC electrolyte, indicating that the weak solvation ability of CMMC contributes to an inorganic-rich SEI, which enables good mechanical properties and fast ion conductivity (Fig. 5b and c, Figs S28–S31). At the same time, weak but distinct C–Cl and LiCl signals were also detected for the CMMC electrolyte (Fig. 5d and e, Figs S28–S31). It is worth noting that the signal intensity of the LiCl peak shows an increasing trend along with the etching depth deepening. Such rigid LiCl bottom structure is conducive to enhancement of the mechanical strength of SEI [46,58]. What’s more, the lower ion diffusion energy barrier of LiCl relative to that of LiF is more beneficial for fast Li^+^ transfer (Fig. 5a). In addition, the high bandgap of LiF is supposed to suppress the excessive reduction of CMMC at the anode side, as well as the possibility of LiCl dissolution in the electrolyte [59].

Regarding the CEI formed on the surface of the NCM811 cathode, a significant amount of Ni was observed for the DMC electrolyte, indicating that the poor CEI layer can’t completely prevent corrosion of the cathode by the electrolyte (Fig. 5g and h). This result can be ascribed to the serious Al corrosion in the DMC electrolyte, as previously demonstrated by the leaking current in Li/Al electrochemical cells (Figs 4b and 5f), causing cracks in the cathode and failure to form a complete CEI. This phenomenon is also proved by plenty of functional groups such as –SO_2_F and –NSO_2_ in N 1s, F 1s and S 2p spectra (Fig. 5i and j, Figs S32–S35), which can be regarded as the corrosion product of the Al current collector [for example, Al(FSI)_3_]. In comparison, the F 1s spectra of the CMMC electrolyte reveals more distinct LiF and C–F peaks, indicating the intact CEI formed by anion and FEC decomposition, which is beneficial for protecting the surface of the NCM811 cathode from corrosion. In addition, the absence of Cl in XPS spectra also reduces the corrosion concerns of Cl-substituted solvent on the cathode side.

### Evaluating safety and electrochemical performance in Ah-level pouch cells

**Figure 6.**
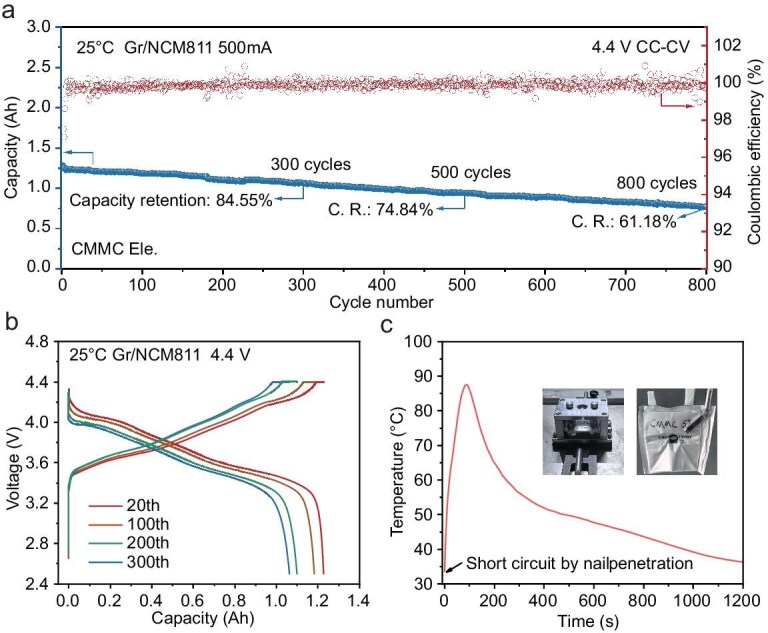
Electrochemical performance and safety evaluation of Ah-level pouch cells. (a) The discharged capacity and CE with cycling number of Gr/NCM811 pouch cells using CMMC Ele. (b) Corresponding charge/discharge profiles. The ∼1.2 Ah pouch cell (2.5–4.4 V) were charged/discharged at 200 mA (∼0.2 C) for two formation cycles and then cycled at 500 mA (∼0.5 C) plus a CC/CV charging procedure with a cut-off current of 100 mA (∼0.1 C). The electrolyte/cathode (E/C) ratio was ∼3.3 g Ah^−1^. (c) Nail penetration experiment and corresponding temperature evolution during the short-circuit process. The insets are cells before (fixed in the instrument) and after nail penetration.

In order to better represent the application interest of Cl-substituted solvent, high-voltage Ah-level Gr/NCM811 pouch cells were further fabricated with the CMMC electrolyte. The batteries showed an initial discharge capacity over 1.2 Ah, corresponding to an energy density of 204.2 Wh kg^−1^ by total weight of cells. Therefore, this cell can satisfy the parameters of commercial applications. We then cycled the pouch cells using 500 mA constant current/constant voltage (CC/CV) charge mode and 500 mA CC discharge mode; 84.55% capacity was still maintained after 300 cycles and the average CE reached 99.84% (Fig. 6a). Impressively, the capacity retention was still 61.18% after 800 cycles, corresponding to a low capacity fading of 0.06% per cycle, demonstrating stable cycling capability at higher voltage compared to the flame-retardant electrolytes reported in the literature (Table S6). Additionally, the charge/discharge curves show slight polarization growth with cycling (Fig. 6b), confirming the stability of the CMMC electrolyte in energy-dense LIBs.

Furthermore, we conducted a nail penetration experiment to verify the safety of our designed electrolyte. The identical electrolyte condition was used as the long-cycling pouch cells mentioned above. The pouch cell was first charged to 100% state-of-charge (SOC), and then forcibly short-circuited by a thick steel needle penetration [60]. Figure 6c shows the temperature evolution during penetration, in which a slow temperature increase rate (∼0.60°C/s) with a peak temperature of 87°C at 88 s was found after the continuous penetration, lower than the boiling temperature of CMMC (139°C–140°C) and decomposition temperature of LiFSI (∼140°C). Moreover, no flames or smoke were observed during the whole penetration experiment (∼1200 s, Videos S3 and S4), proving the high safety of the CMMC electrolyte. The superior performance of CMMC suggests broader opportunities for halogenated electrolytes in advanced LIBs. Beyond the LiFSI salt, the electrolytes with other salts such as LiPF_6_ or LiTFSI also exhibit favorable graphite compatibility (Fig. S36), highlighting the universal applicability of CMMC. Further applying Cl substitution to different conventional solvents (e.g. ethers, cyclic carbonates) is expected to produce more electrolyte formulations suitable for various application scenarios. In addition, the degree of substitution (mono- or multi-Cl) may lead to trade-offs between flame retardancy and electrochemical performance.

## CONCLUSION

In summary, we demonstrate that chlorination in carbonate-based electrolytes can produce LIBs with high safety, high voltage stability and fast reaction kinetics. The binding energy with Li^+^ in Cl-substituted carbonate solvent (CMMC) is 0.11 eV lower than in DMC, which promotes the charge transfer process of the electrochemical reaction, especially at both low temperature and high current density. In addition, the low binding energy of C–Cl is beneficial for the formation of Cl^•^ and reduction at low potential, in which the former provides excellent flame-retardant capability and the latter contributes to generate ionic conductive SEI involving LiCl. Furthermore, due to the fewer free anions in CMMC electrolytes, the corrosion of the Al current collector is largely suppressed with a dense CEI, enabling the operation of NMC811 cathodes at 4.6 V. The Gr/NCM811 1.2 Ah pouch cell exhibited high safety in the nail penetration experiment and revealed a capacity retention over 60% after 800 cycles. This work highlights the chlorine functionality in carbonate-based electrolytes and promotes the development of fluorine-free electrolyte solvents for energy storage/conversion systems.

## Supplementary Material

nwaf420_Supplemental_Files
