# Supplementary material for: Flame-retardant Cl-substituted electrolyte for low-temperature and high-voltage lithium-ion batteries with fast interfacial kinetics
Source: Natl Sci Rev. 2025 Sep 27;13(1):nwaf420. doi: 10.1093/nsr/nwaf420 (PMC12796800; doi:10.1093/nsr/nwaf420)
Supplement: nwaf420_Supplemental_Files [file nwaf420_supplemental_files.zip › NSR-2025-1417-SI.pdf]

Supporting Information

Flame-retardant Cl-substituted electrolyte for low-temperature and high-voltage lithium-ion batteries with fast interfacial kinetics

Yujie Yang[1,2+], Jinyu Zhang[1+], Huaqing Yu[1], Xu Liu[1], Yifei Liu[1], Boyuan Li[1], Jia Li[1], Shuangxin Ren[1], Zhenyu Fan[1], Yawen Li[1], Kun Li[1], Lanqing Wu[1], Qifang Gao[1], Zhenhua Yan[1], Xin Gao[2], Qing Zhao[1*]

[1]Y. Yang, J. Zhang, H. Yu, X. Liu, Y. Liu, B. Li, J. Li, S. Ren, Z. Fan, Y. Li, K. Li, L. Wu. Q. Gao, Prof. Z. Yan, Prof. Q. Zhao, State Key Laboratory of Advanced Chemical Power Sources, Frontiers Science Center for New Organic Matter, Key Laboratory of Advanced Energy Materials Chemistry (Ministry of Education), College of Chemistry, Nankai University, Tianjin, 300071, China.

[2]Y. Yang, Prof. X. Gao, School of Materials Science and Engineering, Peking University, Beijing, 100871, China; Center for Nanoscale Science and Technology, Academy for Advanced Interdisciplinary Studies, Peking University, Beijing, 100871, China.

***Corresponding author.** E-mail: zhaoq@nankai.edu.cn

[+]These authors contribute equally to this work.

Reagents and materials

The reagents used in this work include chloromethyl methyl carbonate (CMMC, 98%, Aladdin), dimethyl carbonate (DMC, ≥99%, Aladdin), fluoroethylene carbonate (FEC, ＞98.0%, TCI), lithium bis(fluorosulfonyl)amide (LiFSI, 99.9%, DoDoChem). All the solvents were dried with molecular sieves before use.

The graphite anodes (average areal capacity: 0.6 mAh cm^{-2}) used in the Li/Gr half cells were self-made as follows: graphite active material, Super P, and PVDF were mixed in a mass ratio of 8:1:1 and dispersed in N-Methylpyrrolidone (NMP, 99.9%, Aladdin) to make a slurry. The slurry was coated onto copper foil current collectors, followed by drying in a vacuum oven at 120 °C for 10 hours. All the NCM811 cathodes (1.56 mAh cm^{-2}) in this work, the graphite anodes (1.66 mAh cm^{-2}) used in the Gr/NCM811 full cells, the Li metal (Φ14 mm×0.4 mm), and the 1 Ah dry pouch cell (68 mm×58 mm, packed with spirally wound method) were purchased from Guangdong Canrd New Energy Technology Co. LTD. The areal capacity of cathode and anode in the pouch cell is 2.68 and 3.24 mAh cm^{-2}, respectively.

Preparation of Electrolytes and Cells

All electrolyte preparation and CR2032 coin cell assembly were conducted in an argon-filled glove box with rigorously controlled atmospheric condition (H_2O<0.1 ppm, O_2<2.0 ppm). The specific formulations of the prepared electrolytes are detailed in **Table S1**. The electrolyte usage for each coin cell was 40 μL and the separator is Al_2O_3 coated Celgard. The dry pouch cell, after electrolyte injection in the glove box, was transferred to a climate-controlled dry room (Drew point: around -50 °C) for vacuum sealing. The sealed pouch cell underwent formation and degassing processes before subsequent electrochemical testing.

Electrochemical tests

Galvanostatic charge/discharge measurements were conducted on the batteries using a LAND CT3002A test system to evaluate their electrochemical performance. Specifically, before conducting low-temperature (-20°C) electrochemical performance tests, the coin cells were first activated through five cycles at 0.2 C under room temperature, followed by a final lithiation step to ensure the full lithium intercalation state of graphite anode. The constant voltage charging test of Li/Al cells at 4.5 V was also conducted on the LAND CT3002A test system. All batteries employing NCM811 cathodes, as well as Li/Al cells used for LSV and constant voltage tests, were assembled with aluminum-plated positive electrode casings to mitigate the corrosive effects of LiFSI on stainless steel. Voltage floating test in Li/NCM811 cells was

operated first by galvanostatic charge to 4.3 V at 0.1 C rate, then the voltage was held for 10 hours and gradually increase with 0.1 V step for 10 hours each step until the voltage reach 4.6 V. The pouch cells underwent formation process before long-term cycling. After electrolyte injection into dry pouch cells, they were subjected to a 12 h resting period, sealed in a dry room, and then underwent the following electrochemical formation procedure: 50 mA charge for 1 h (or voltage ≥ 3.6V) - 100 mA charge for 6 h (or voltage ≥ 4.0 V) - 200 mA charge for 5 h (or voltage ≥ 4.3 V) - 30 min rest - 330 mA discharge to voltage ≤ 2.5 V - 330 mA charge for 3 h (or voltage ≥ 3.65 V). Then they were degassed to remove gases generated during formation, followed by final sealing for standard long-term cycling tests.

An electrochemical workstation (CHI 760E) was used to conduct electrochemical impedance spectroscopy (EIS) in the frequency range of 0.1-10000 Hz and linear sweep voltammetry (LSV) at a scan rate of 1 mV/s. The ionic conductivity of the electrolytes at different temperatures was determined from EIS measurements using the equation $\sigma = \frac{L}{R\times S}$, where R represents the electrolyte resistance measured in a stainless steel-blocked cylindrical cell with defined geometric parameters (area: S, hight: L). The energy barriers associated with Li^+ charge transfer (lithium-ion desolvation) and crossing SEI were quantitatively determined through Arrhenius equation analysis ($\frac{1}{R} = \frac{1}{R_0} e^{-E_a/K_B T}$) based on the electrochemical impedance spectroscopy (EIS) data obtained from graphite|electrolyte|graphite symmetric cells.

The Li^+ transference number of Li/Li cells was determined using potentiostatic polarization method on a CHI 760e electrochemical workstation. A potential step of 5 mV was applied to the cell for 3600 s to record the initial current and the steady-state current. Electrochemical impedance spectroscopy (EIS) measurements were performed both before and after polarization to determine the initial and final cell impedances. The Li^+ transference number was calculated according to:

$$t_+ = \frac{I_s(\Delta V - I_0 R_0)}{I_0(\Delta V - I_s R_s)}$$

Where I_s is the steady-state current, I_0 is the initial current, R_0 is the initial impedance, R_s is the final impedance, ΔV is the applied potential.

Characterizations

The graphite samples were subjected to comprehensive surface analysis using field-emission scanning electron microscopy (SEM, JEOL JSM7900F; JEOL, Tokyo, Japan) coupled with energy-dispersive X-ray spectroscopy (EDS) to investigate both the morphological features and elemental distribution characteristics. For X-ray diffraction (XRD, SmartLab 9KW, Rigaku

Corporation) characterization, the graphite samples were prepared in an glove box and hermetically sealed with paraffin film to prevent air exposure. The sample preparation for $^{7}Li/^{17}O$ NMR (AVANCE III 400MHz, Bruker) characterization was performed as follows: (1) a 1 M LiCl in D_2O standard solution was sealed in a glass capillary and (2) this capillary was then inserted into a NMR tube containing 500 μL electrolyte. The infrared (IR) spectra of the electrolytes were acquired in attenuated total reflection (ATR) mode using a Fourier transform infrared (FT-IR) spectrometer (TENSOR II, Bruker, Billerica, USA). Thermal analysis of the electrolytes was conducted using differential scanning calorimetry (DSC 214 Ployma, NETZSCH) with a controlled heating/cooling rate of 10 °C/min from 60 °C to 150 °C. To analyze the composition of solid electrolyte interphases on both electrodes, Gr/NCM811 full cells were assembled and subjected to five charge-discharge cycles at room temperature. Subsequently, the cells were disassembled in glove box and the electrodes were carefully taken out. The electrode surfaces were then rinsed with dimethyl ether (DME) to remove residual electrolyte, followed by complete DME evaporation prior to X-ray photoelectron spectroscopy (XPS) characterization. The XPS analysis was performed using an Escalab 250Xi X-ray photoelectron spectrometer (ThermoFisher Scientific) equipped with a monochromatic Al Kα X-ray source. All elements were calibrated according to the reference value 284.6 eV for adsorbed carbon

Flammability test

The flammability of electrolytes was evaluated by introducing 500 μL of DMC or CMMC solvent into a stainless steel battery casing, followed by ignition using a lighter. The combustion process was recorded with a camera, and images were captured at key stages to comparatively assess the flame-retardant properties of different solvents. The pouch cells (same as those for long-cycle test) were charged to 100% state of charge (SOC) to conduct the nail penetration short-circuit test with an 8-mm-diameter steel needle at a penetration speed of 75 mm/s using an integrated nail penetration/crush testing machine (MSK-TE905-UL). The needle was maintained in the penetrated position throughout the test to ensure persistent short-circuit conditions.

Theoretical Calculations

Theoretical calculations: The molecular structures were first optimized at the level of B3LYP/6-31G+(d, p) to find the lowest energy structure and refined at the level of M062X/6-311G+(d, p). Gaussian 16 software package and Gaussview 6.0 were used for the DFT calculations and

the visualization of molecular structures, respectively [1,2]. Based on the optimized results, the ESP of the solvent was analyzed by using Multiwfn software [3]. To calculate the binding energy, Li ion was introduced at the position where the electrostatic potential is the most negative and the structure was reoptimized for each configuration. The HOMO/LUMO calculations were conducted in the gas phase and based on M062X/6-311G+(d, p). In addition, the bond energy was defined as follows:

$$E = E_{x-y} - E_x - E_y$$

where E_{x-y} is the energy of solvent and E_x and E_y are the energies of free radicals x and y, respectively.

Molecular dynamics simulations were carried out using GROMACS 2018 [4,5]. The force field parameters for solvents and ions were derived from the general AMBER force field (GAFF) [6,7]. The corresponding atomic charges were generated from the restrained electrostatic potential RESP charges [8,9], using Gaussian 16 software package. The atomic charges of ions were scaled down by a factor of 0.8 to account for charge polarization. The simulation procedure consisted of an energy minimization using the steepest descent method followed by a 3 ns equilibration step in the NPT ensemble and a 20 ns production run using NVT ensemble. The temperature was maintained at 298 K using V-rescale thermostat, while the pressure was maintained at 1 bar using Berendsen barostat. The cutoffs for the short-range electrostatic and van der Waals interactions were set to 1.2 nm. Periodic boundary conditions were applied in all directions. The convergence of the system energy, temperature and box size were checked to verify equilibration state. The radial distribution functions (RDFs) were calculated using GROMACS. The coordination distance was defined as the position of the first peak in the RDF, and the coordination number was determined as the value of the coordination number curve at the first minimum following the first peak. The VMD software was used to visualize the snapshot of the MD simulation [10]. The 3D trajectory of the Li ions was represented by calculating the average position of the Li ions, and the 3D trajectory of the FSI ions was represented by calculating the average centroid position of the FSI ions, and x, y, and z are the simulated 3D position coordinates of a specific time point, respectively. To visualize the movement trajectory, Li ions and FSI ions were plotted across thirteen consecutive time frames, and each frame was separated by a simulation interval of 40 picoseconds. The average displacement of Li ions was calculated by averaging the displacement of each Li ion between consecutive time points. The average distance between Li ions and FSI ions was calculated by averaging the distances between their trajectory positions at each time point.

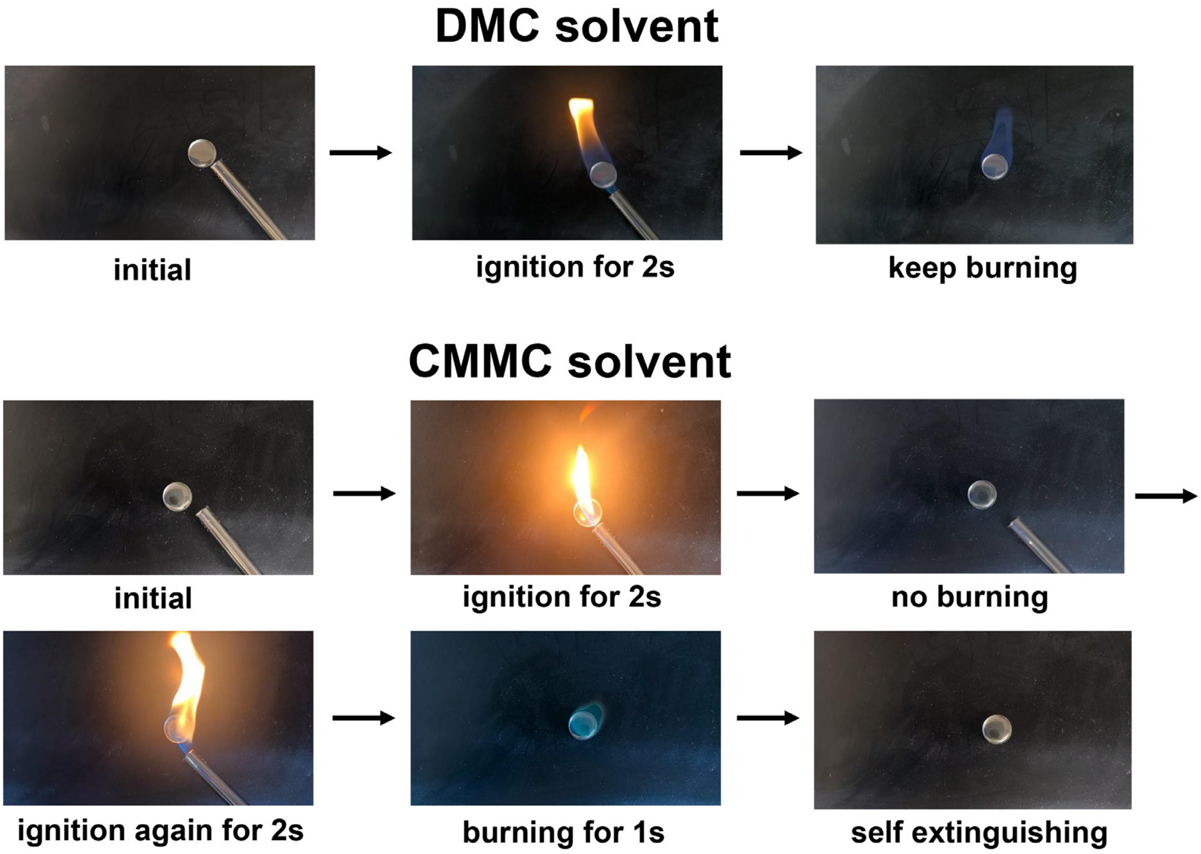

Figure S1. Ignition experiment of (a) DMC solvent and (b) CMMC solvent.

Table S1. Composition of the experimental electrolytes

**Electrolytes**	**Formulation**
CMMC Ele. W/O FEC	1 mmol LiFSI in 1 mL CMMC
CMMC Ele.	1 mmol LiFSI in 950 μL CMMC and 50 μL FEC
DMC Ele. W/O FEC	1 mmol LiFSI in 1 mL DMC
DMC Ele.	1 mmol LiFSI in 950 μL DMC and 50 μL FEC
CMMC Ele. (2% FEC)	1 mmol LiFSI in 980 μL DMC and 20 μL FEC
CMMC Ele. (10% FEC)	1 mmol LiFSI in 900 μL DMC and 100 μL FEC
CMMC Ele. (with $LiPF_6$)	1 mmol $LiPF_6$ in 950 μL CMMC and 50 μL FEC
CMMC Ele. (with LiTFSI)	1 mmol LiTFSI in 950 μL CMMC and 50 μL FEC

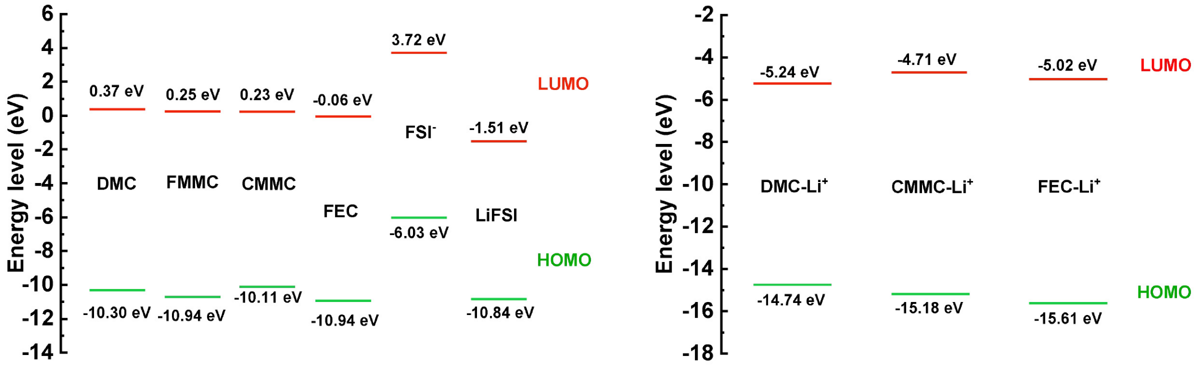

Figure S2. Calculated HOMO and LUMO energy levels for both isolated solvents or anion and their Li^+-coordinated complexes.

Table S2. Frontier orbital energies of different components (eV)

Chemical composition	HOMO	LUMO	GAP
DMC	-10.30	0.37	10.67
FMMC	-10.72	0.25	10.97
CMMC	-10.11	0.23	10.34
FEC	-10.94	-0.06	10.88
FSI^-	-6.03	3.72	9.75
LiFSI	-10.84	-1.51	9.33

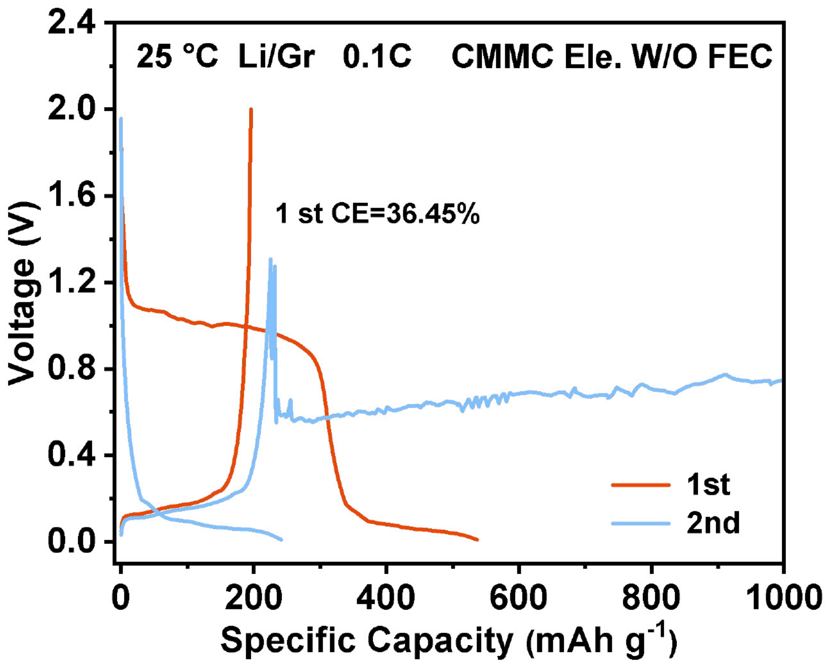

Figure S3. Initial charge/discharge profiles and second-cycle failure profiles of Li/Gr half cell using CMMC Ele. W/O FEC.

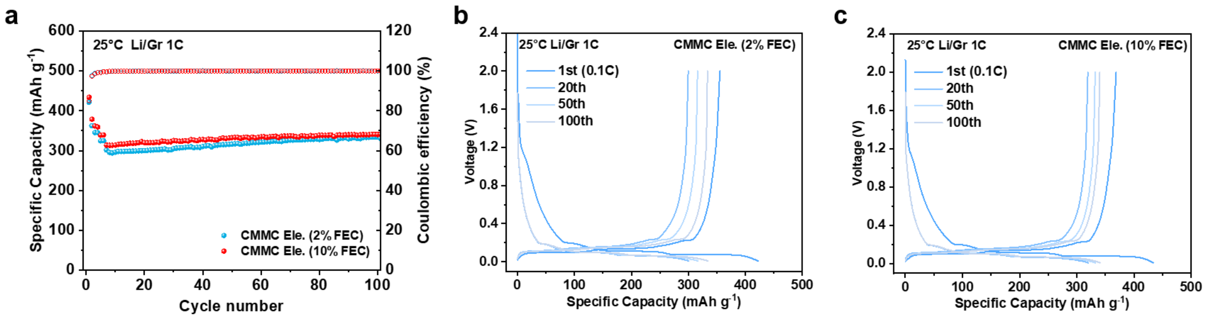

Figure S4. (a) Specific capacity and Coulombic efficiency of the Li/Gr half cell with different content of FEC. The corresponding galvanostatic discharge/charge profiles of (b) 2% FEC and (c) 10% FEC at different cycles.

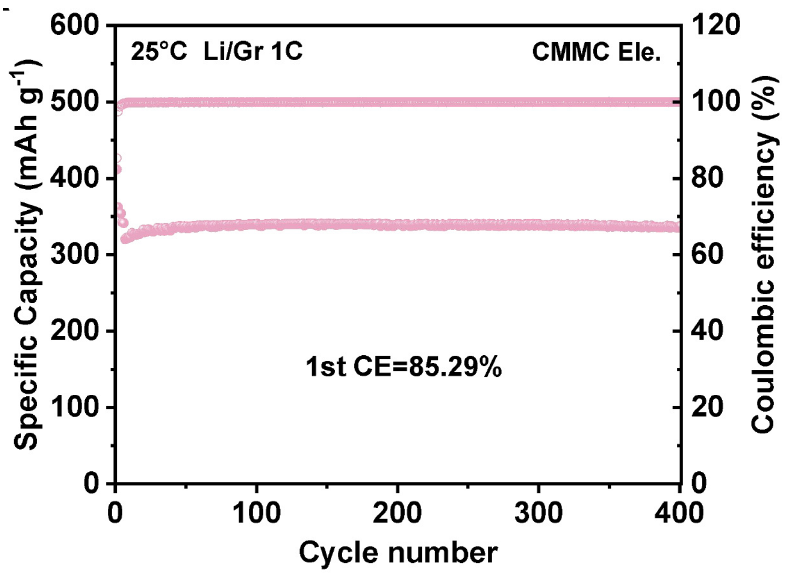

Figure S5. Specific capacity and Coulombic efficiency of the Li/Gr half cell using CMMC Ele. at 1 C. The capacity retention at the 400th cycle is calculated relative to the stabilized discharge capacity at the 5th cycle.

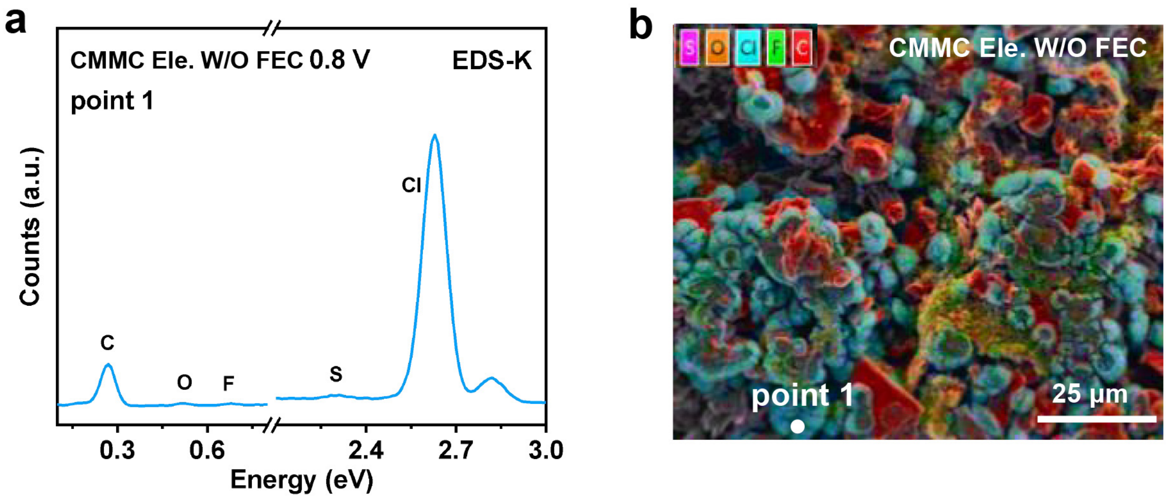

Figure S6. (a) SEM EDS spectra of graphite electrode located at Point 1 marked in (b). The graphite electrode is discharged to 0.8 V using CMMC Ele. W/O FEC.

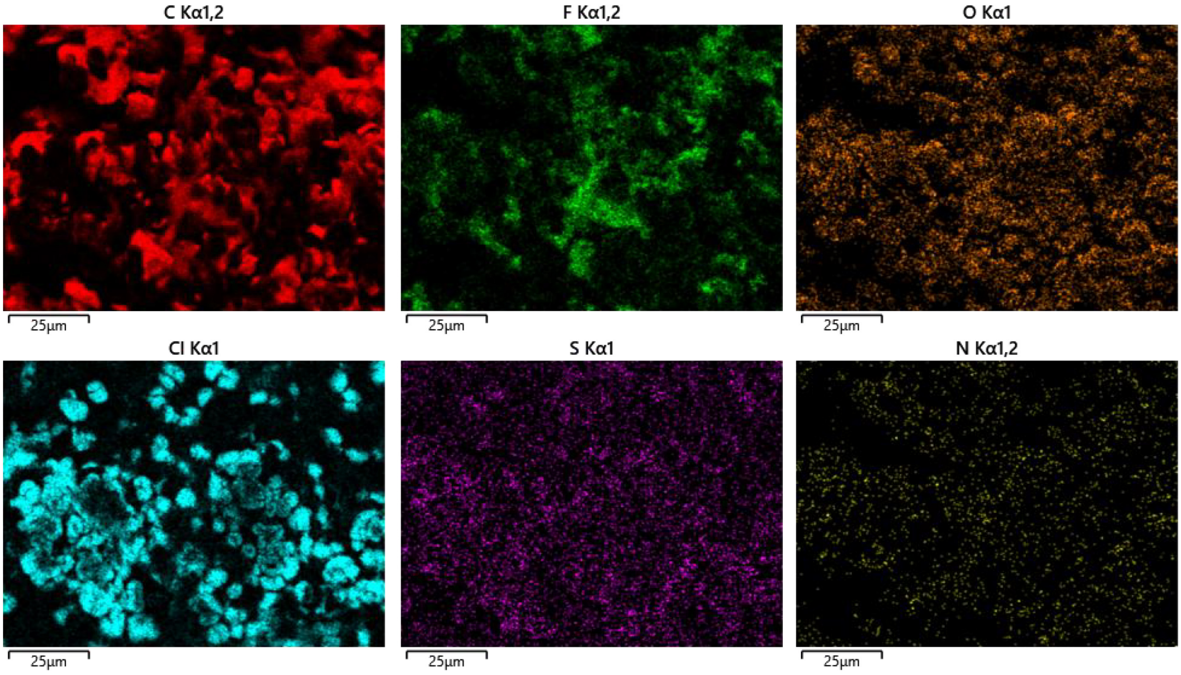

Figure S7. SEM-EDS C, F, O, Cl, S, N element mapping of graphite surface using CMMC Ele. W/O FEC.

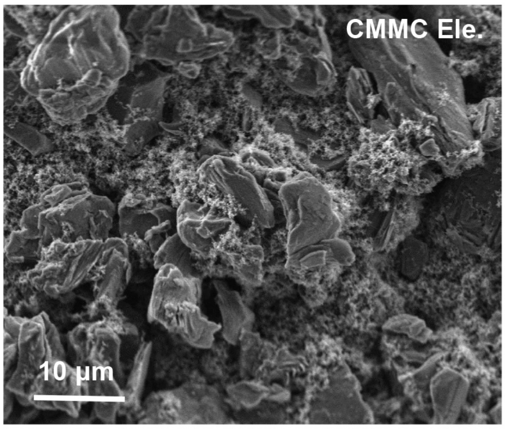

Figure S8. SEM morphology of graphite electrodes discharged to 0.8 V using CMMC Ele..

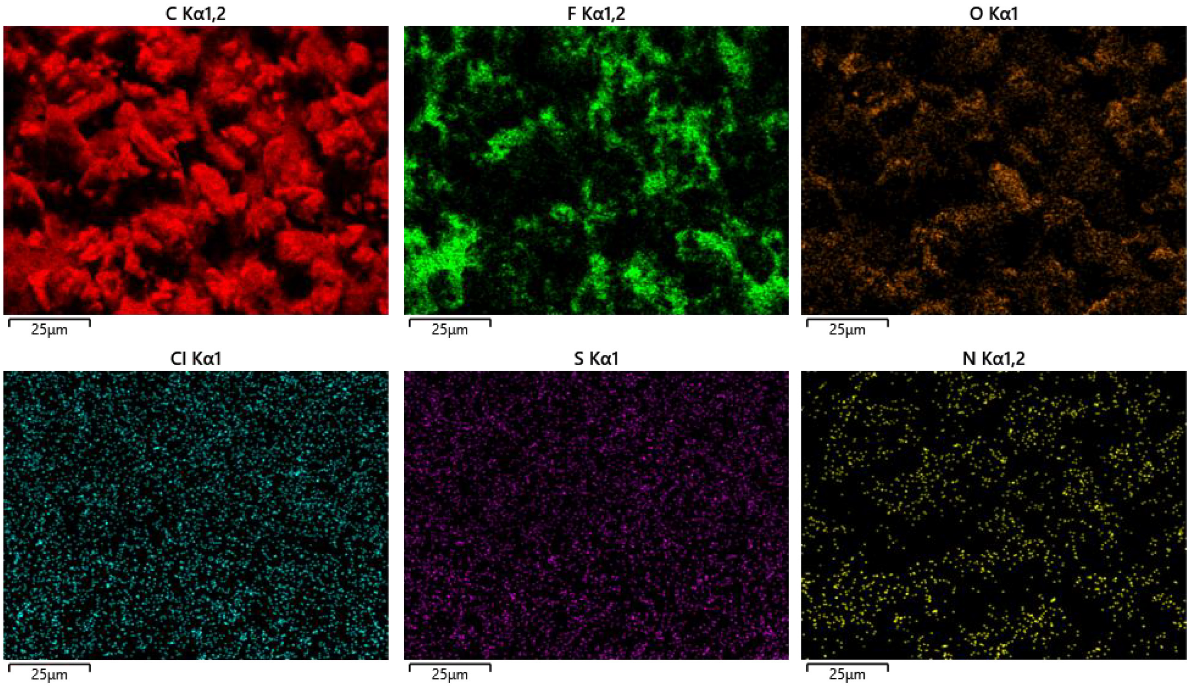

Figure S9. SEM-EDS C, F, O, Cl, S, N element mapping of graphite surface using CMMC Ele..

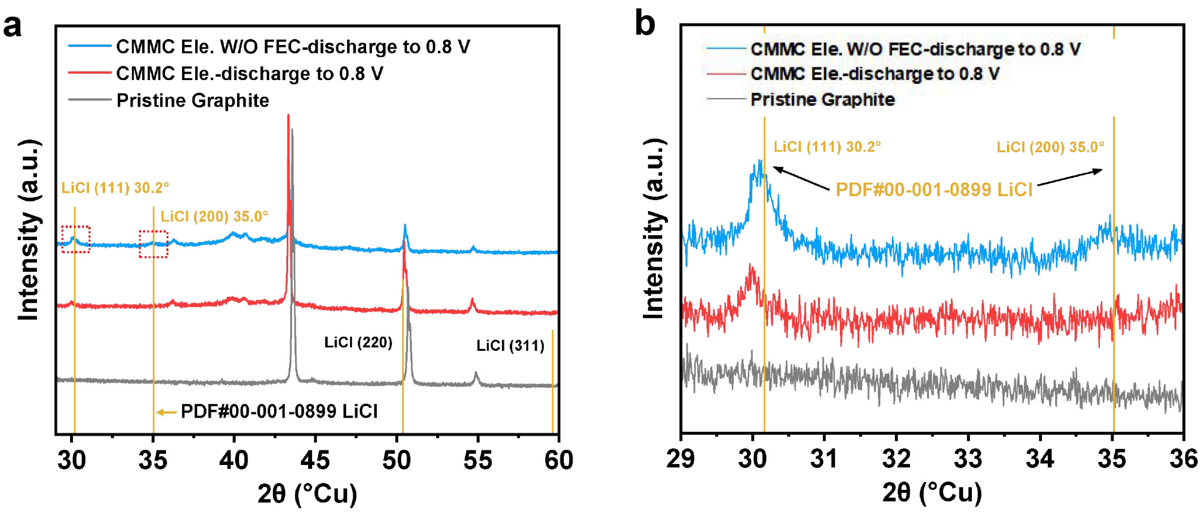

Figure S10. (a) XRD pattern and (b) localized magnification of the graphite electrodes discharged to 0.8 V using CMMC Ele. and CMMC Ele. W/O FEC. The pristine graphite electrode was also tested as comparison.

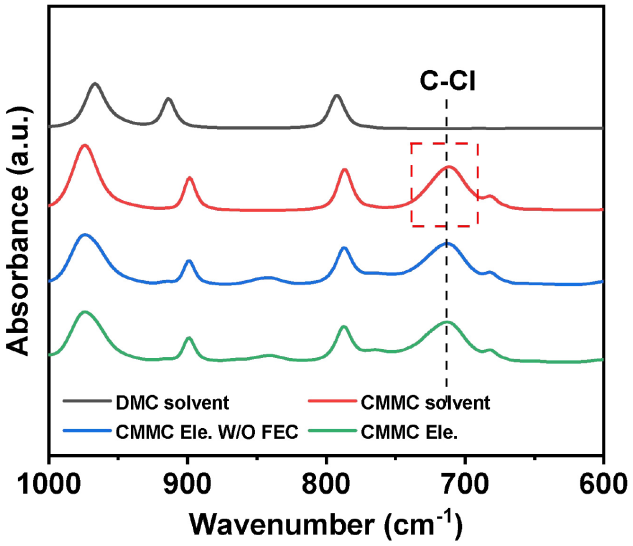

Figure S11. ATR-FTIR spectral characterizations of CMMC Ele. and CMMC Ele. W/O FEC. Pure DMC and CMMC solvent were also tested for comparison.

Table S3. The coordination number of Li^+ in different electrolytes

Electrolytes	Li^+-DMC	Li^+-FEC	Li^+-FSI^-	Li^+-CMMC
DMC Ele.	3.91	0.26	0.70	---
CMMC Ele.	---	0.55	1.88	2.86

Table S4. The coordination distance of Li^+ in different electrolytes

Electrolytes	Li^+-DMC	Li^+-FEC	Li^+-FSI^-	Li^+-CMMC
DMC Ele.	2.02 Å	2.08 Å	2.16 Å	---
CMMC Ele.	---	2.04 Å	2.12 Å	2.04 Å

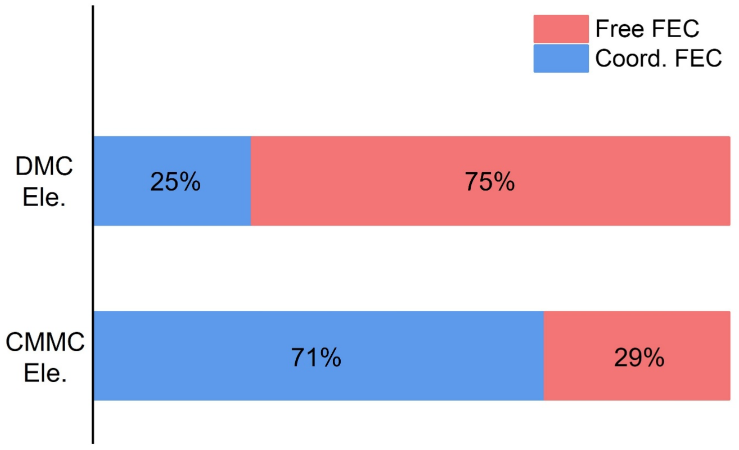

Figure S12. Ratio of free FEC and coordinated FEC in studied electrolytes.

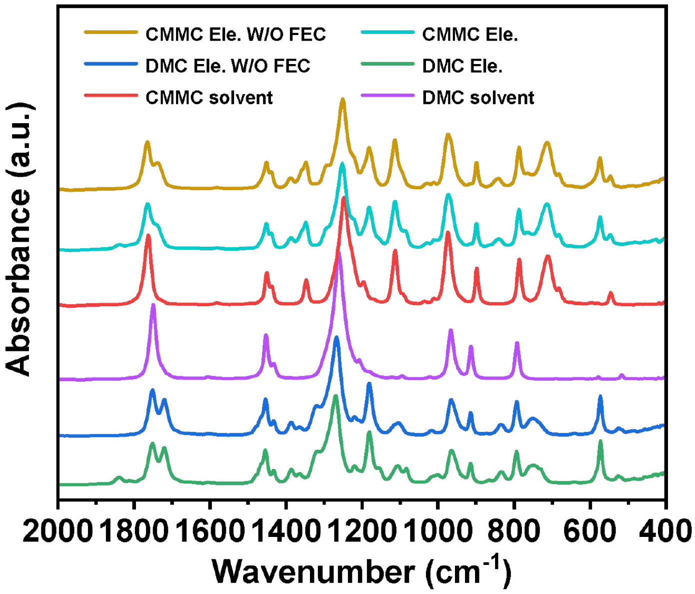

Figure S13. ATR-FTIR spectral characterizations of CMMC Ele. W/O FEC, CMMC Ele., DMC Ele. W/O FEC and DMC Ele.. Pure CMMC and DMC solvent were also tested for comparison.

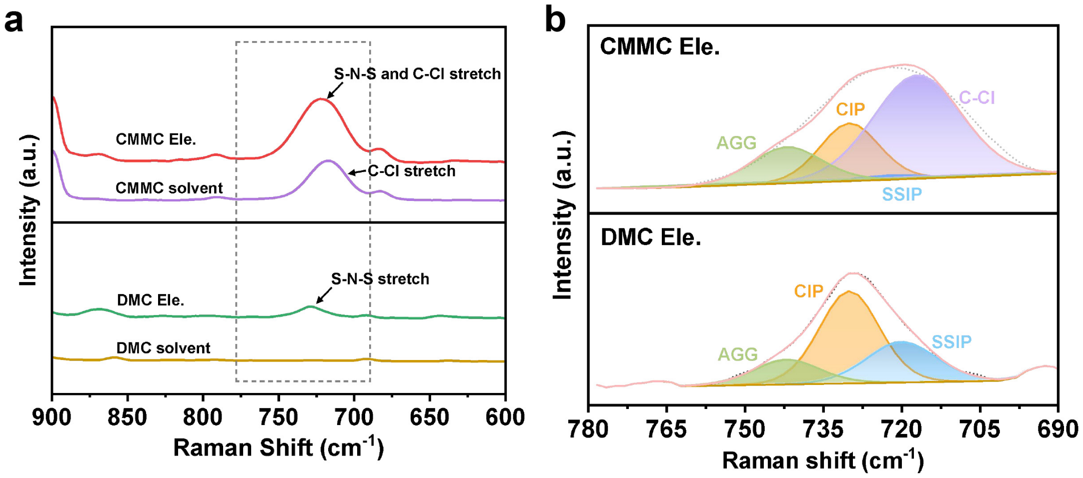

Figure S14. (a) Raman spectra of CMMC Ele. and DMC Ele.. Pure CMMC and DMC solvent were also tested for comparison. (b) The distribution of AGG, CIP, and SSIP in different electrolytes.

It should be noted that the C-Cl bond peak of the CMMC solvent appears near 717 cm^{-1}, which may interfere with the peak analysis of the SSIP structure.

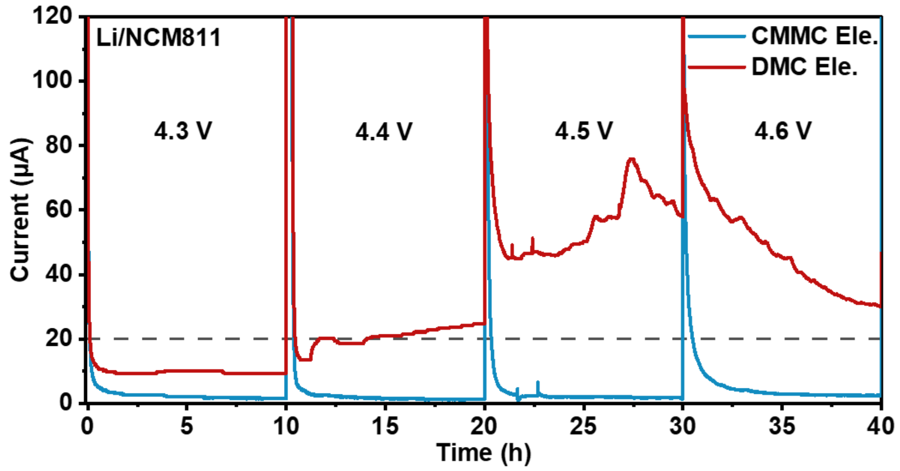

Figure S15. Constant-voltage floating test in Li/NCM811 cells for oxidation stability evaluation from 4.3 V to 4.6 V.

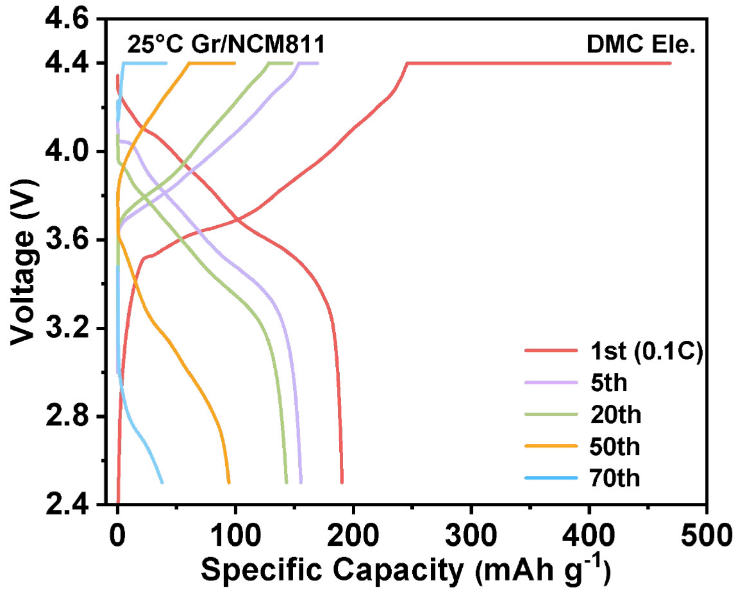

Figure S16. Galvanostatic discharge/charge profiles of DMC Ele. at different cycles at 0.5 C and 4.4 V cut-off voltage.

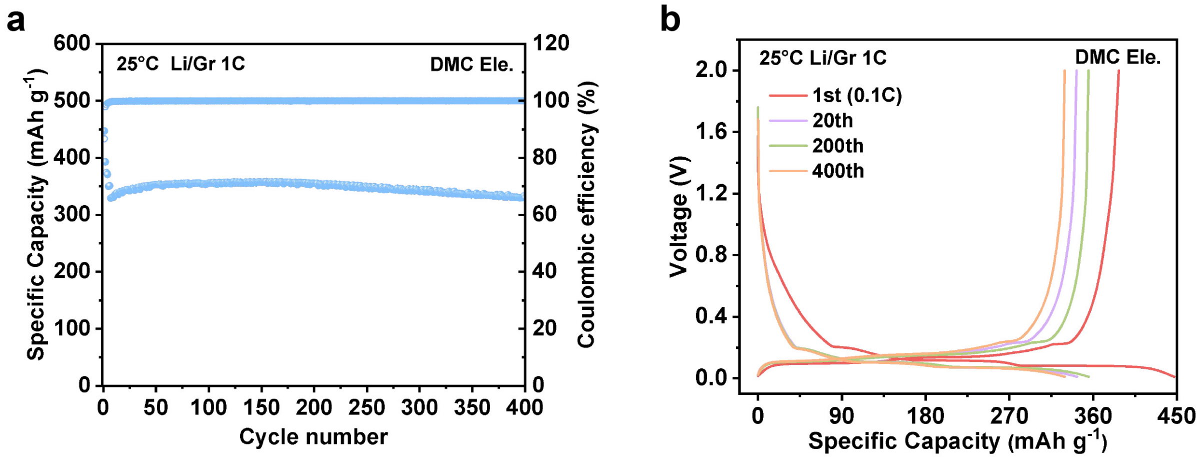

Figure S17. (a) Specific capacity and Coulombic efficiency of the Li/Gr half cell using DMC Ele. at 1 C. (b) The corresponding galvanostatic discharge/charge profiles at different cycles.

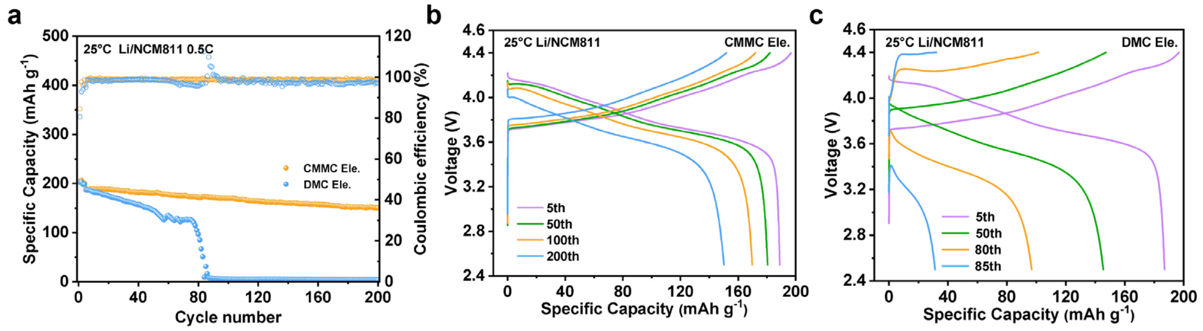

Figure S18. (a) Cycle performance of Li/NCM811 half cells at 0.5 C and 4.4 V cut-off voltage. Corresponding galvanostatic discharge/charge profiles of (b) CMMC Ele. and (c) DMC Ele. at different cycles.

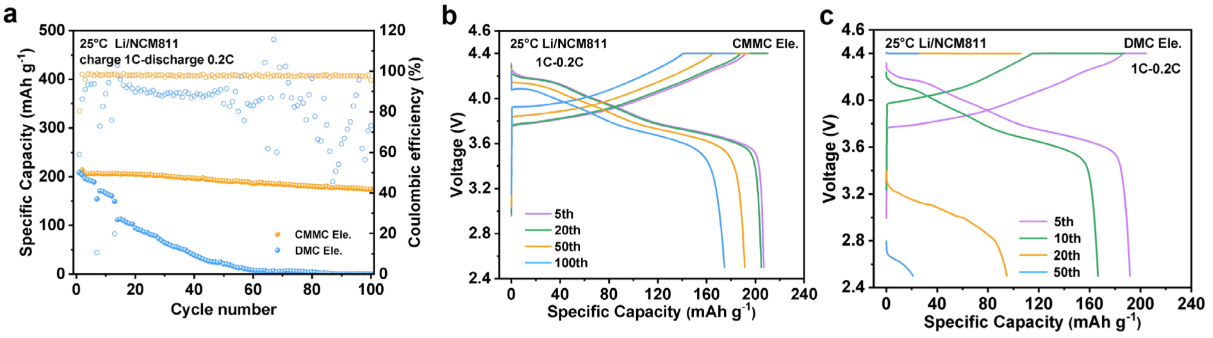

Figure S19. (a) Cycle performance of Li/NCM811 half cells at 1 C charging-0.2 C discharging and 4.4 V cut-off voltage. Corresponding galvanostatic discharge/charge profiles of (b) CMMC Ele. and (c) DMC Ele. at different cycles. The cells were charged/discharged at 0.1 C for two formation cycles and then cycled at 1 C plus constant voltage (CC-CV) charging procedure with a cut-off current of 0.1 C.

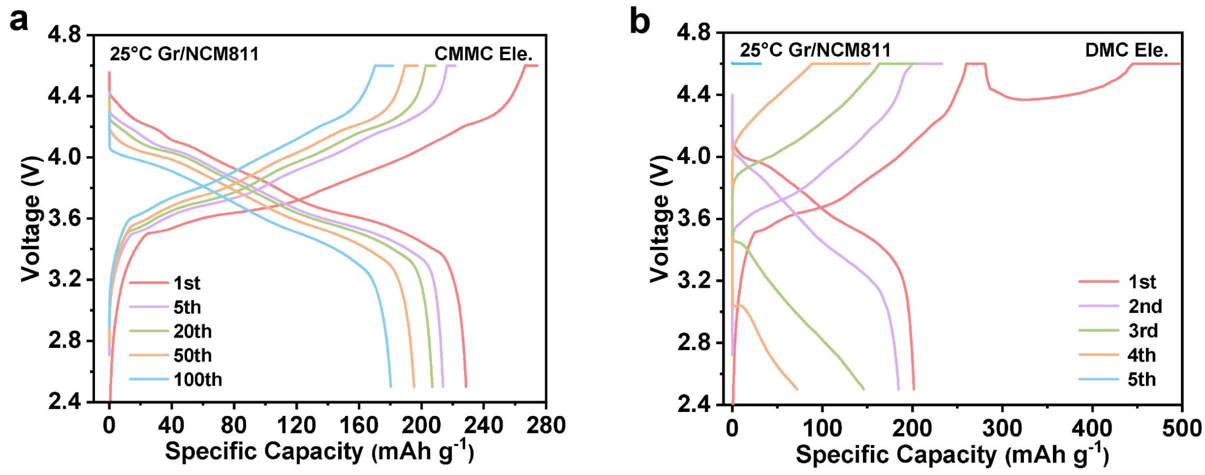

Figure S20. Corresponding galvanostatic discharge/charge profiles of the Gr/NMC811 full cells at 4.6 V using (a) CMMC Ele. and (b) DMC Ele..

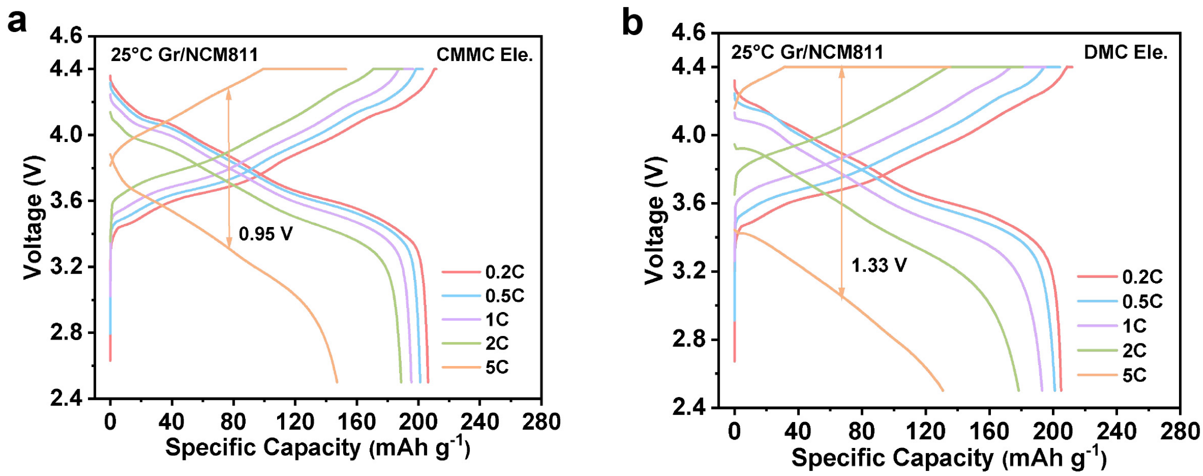

Figure S21. Corresponding galvanostatic discharge/charge profiles and polarization potential at 5 C of the Gr/NMC811 full cells in rate testing using (a) CMMC Ele. and (b) DMC Ele..

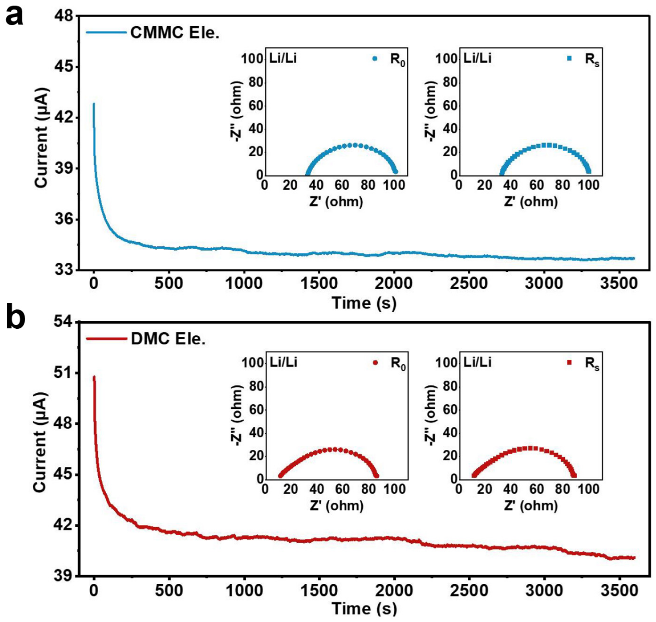

Figure S22. Li^+ transference number measurement of (a) CMMC electrolyte and (b) DMC electrolyte.

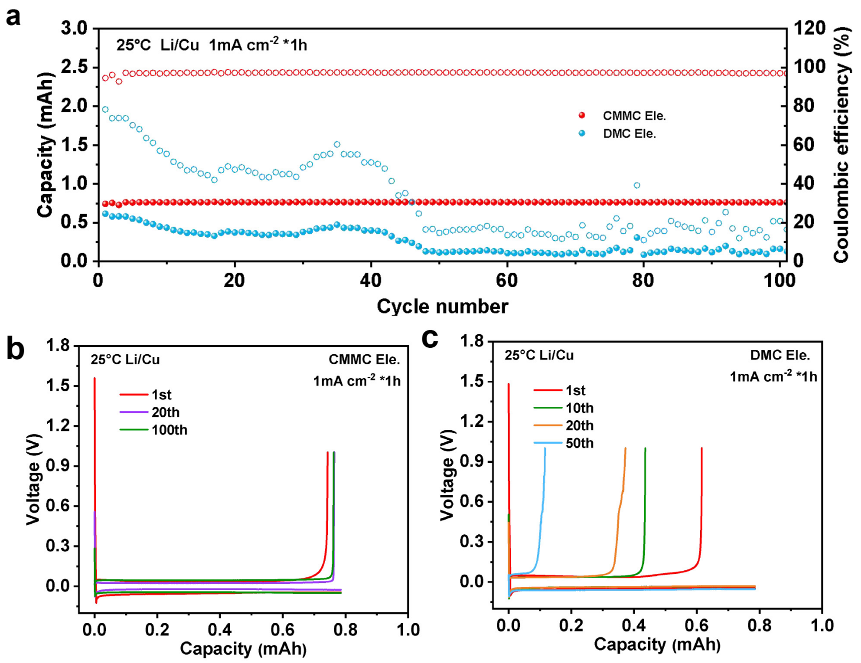

Figure S23. (a) Cycling performance of Li/Cu cells using CMMC Ele. and DMC Ele.. Corresponding galvanostatic discharge/charge profiles of (b) CMMC Ele. and (c) DMC Ele. at different cycles.

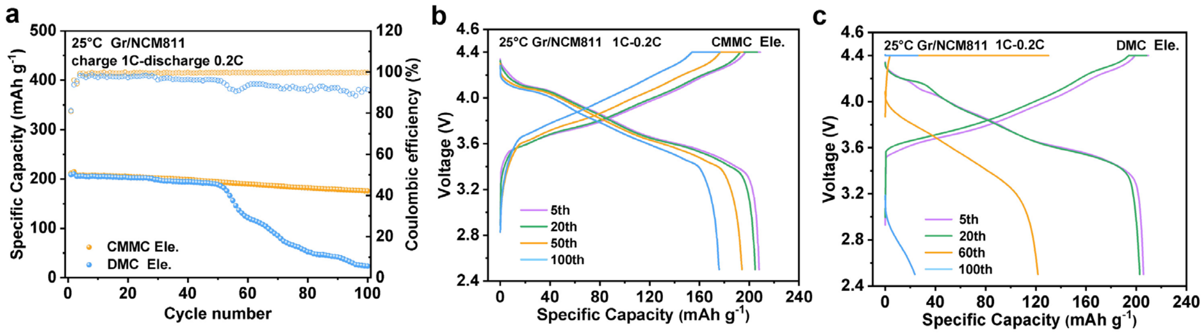

Figure S24. (a) Cycle performance of Gr/NCM811 full cells at 1 C charging-0.2 C discharging and 4.4 V cut-off voltage. Corresponding galvanostatic discharge/charge profiles of (b) CMMC Ele. and (c) DMC Ele. at different cycles. The cells were charged/discharged at 0.1 C for two formation cycles and then cycled at 1 C plus constant voltage (CC-CV) charging procedure with a cut-off current of 0.1 C.

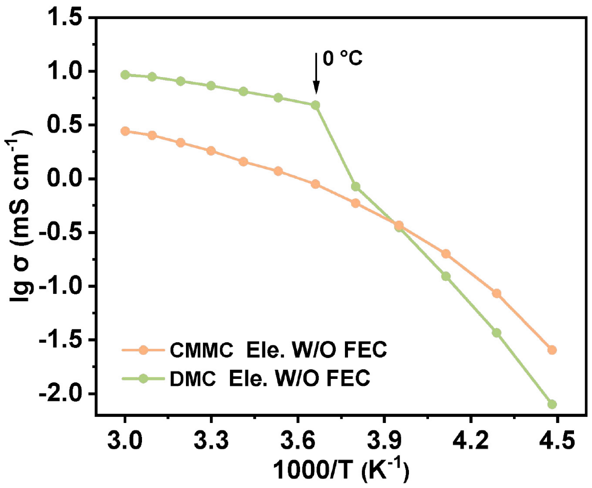

Figure S25. Ionic conductivity versus temperature of CMMC Ele. W/O FEC and DMC Ele. W/O FEC.

Table S5. Ionic conductivity of the electrolytes (mS cm^{-1})

	**CMMC Ele. W/O FEC**	**CMMC Ele.**	**DMC Ele. W/O FEC**	**DMC Ele.**
**60 °C**	2.77	3.88	9.26	10.84
**50 °C**	2.53	3.33	8.85	10.03
**40 °C**	2.16	2.89	8.08	9.13
**30 °C**	1.82	2.44	7.31	8.17
**20 °C**	1.44	2.03	6.48	7.13
**10 °C**	1.17	1.62	5.66	6.23
**0 °C**	0.89	1.19	4.84	5.23
**-10 °C**	0.59	0.82	0.85	1.60
**-20 °C**	0.40	0.47	0.35	0.58
**-30 °C**	0.20	0.24	0.13	0.19
**-40 °C**	0.09	0.12	0.04	0.06
**-50 °C**	0.04	0.04	0.02	0.04

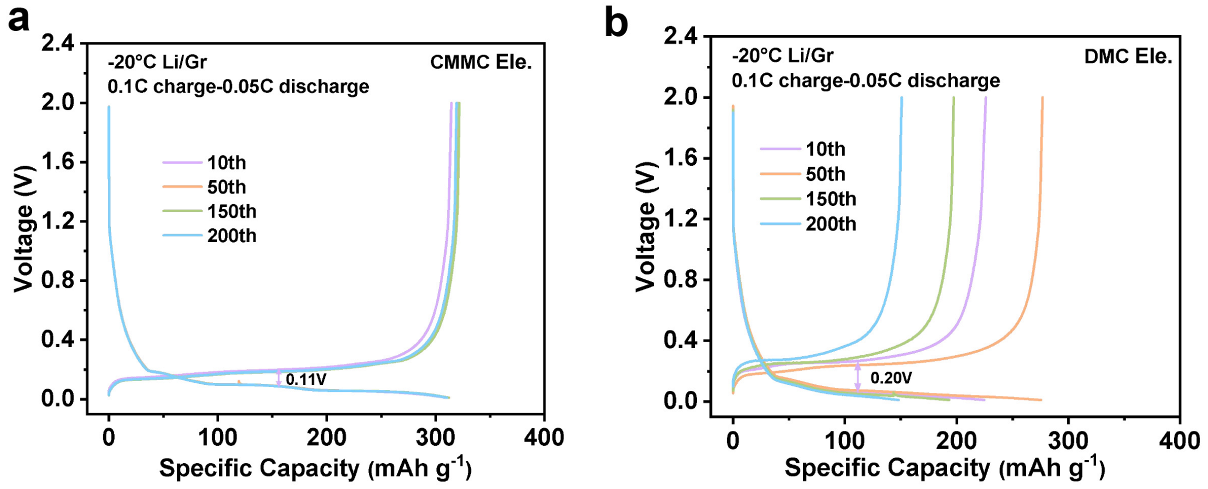

Figure S26. Discharge/charge profiles and polarization potential at the 10th cycle of the Li/Gr half cells at -20 °C using (a) CMMC Ele. and (b) DMC Ele..

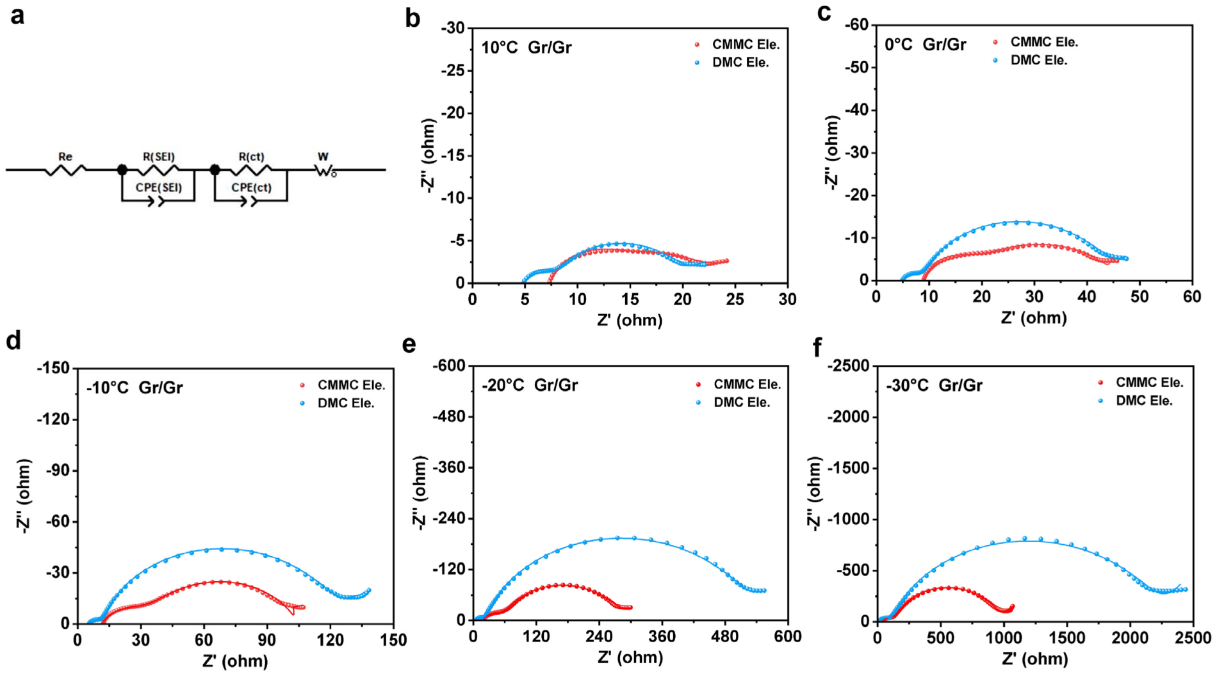

Figure S27. (a) The equivalent circuit for electrochemical impedance spectra. EIS spectra of CMMC Ele. and DMC Ele. at b) 10 °C, c) 0 °C, d) -10 °C, e) -20 °C, and f) -30 °C.

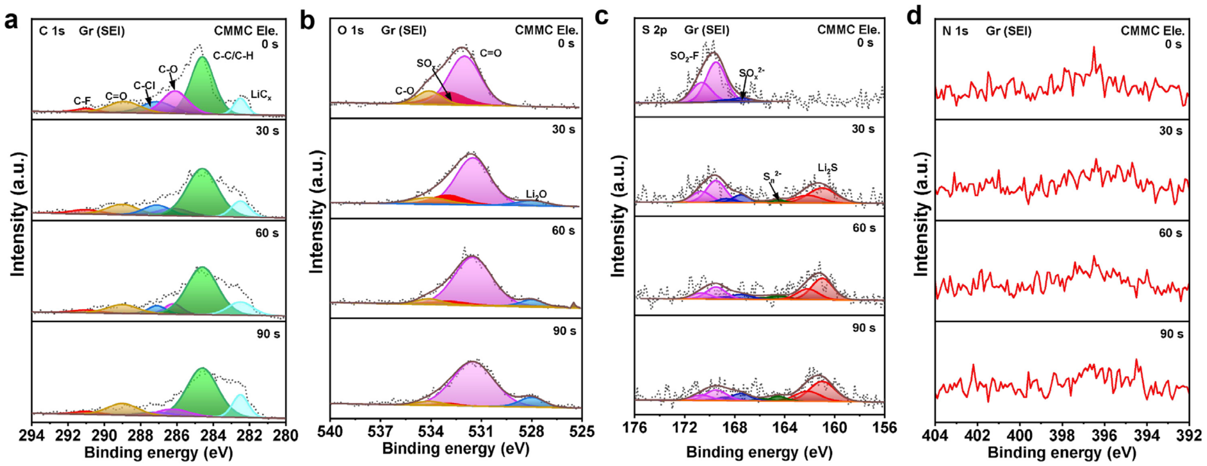

Figure S28. (a) C 1s, (b) O 1s, (c) S 2p, and d) N 1s XPS fine spectra of graphite anode in CMMC Ele.. All elements have been calibrated according to the reference value 284.6 eV for adsorbed carbon.

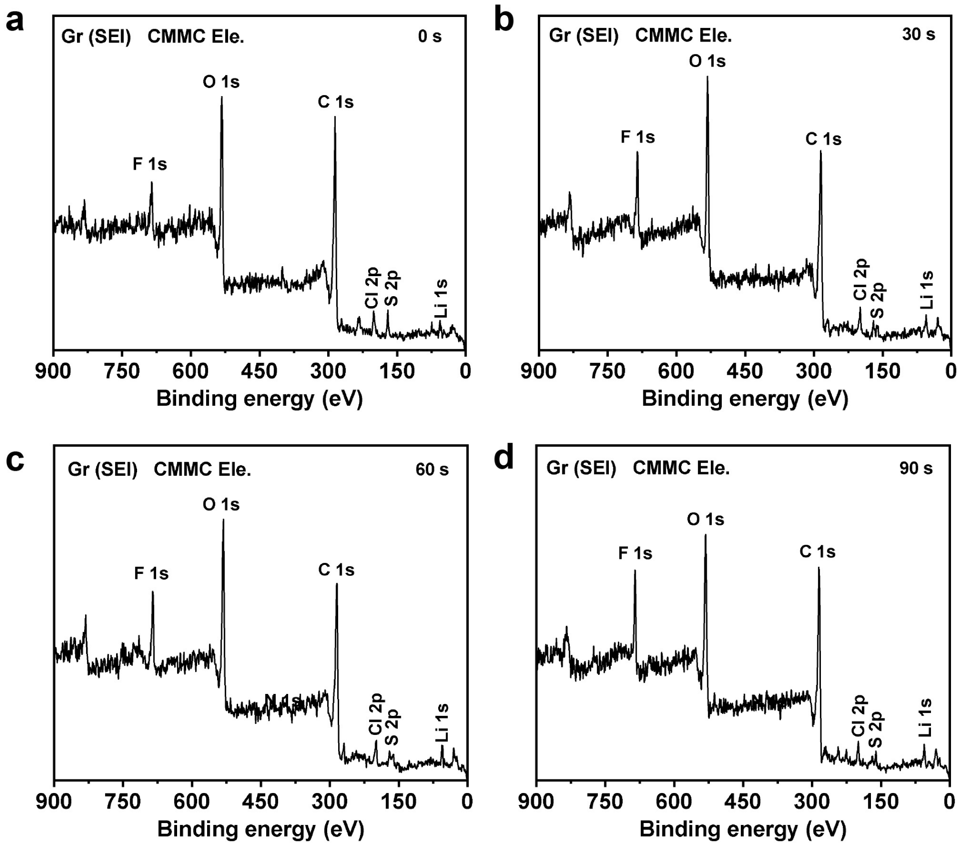

Figure S29. XPS full spectra of graphite anode cycled in CMMC Ele..

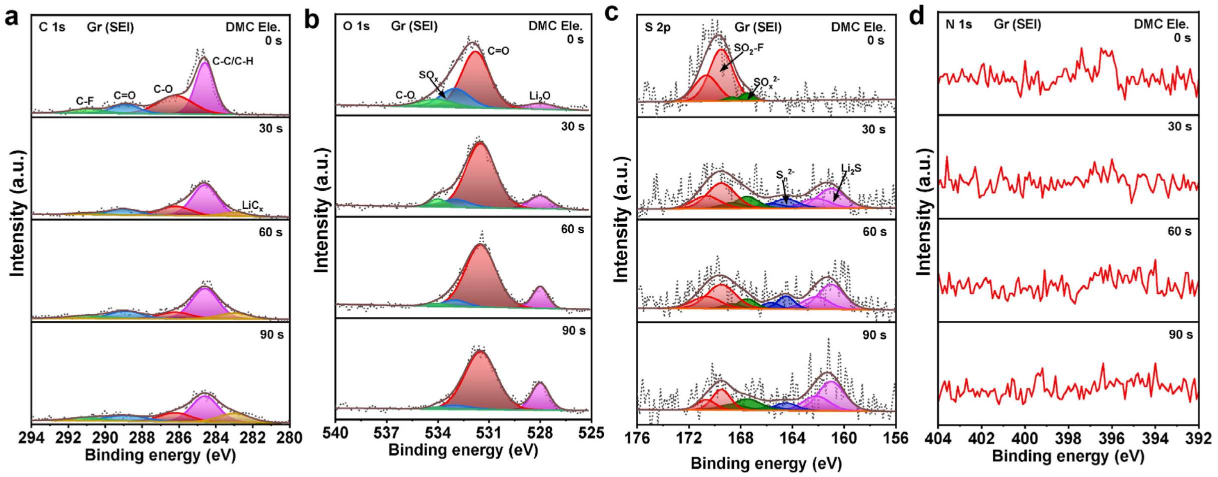

Figure S30. (a) C 1s, (b) O 1s, (c) S 2p, and d) N 1s XPS fine spectra of graphite anode in DMC Ele.. All elements have been calibrated according to the reference value 284.6 eV for adsorbed carbon.

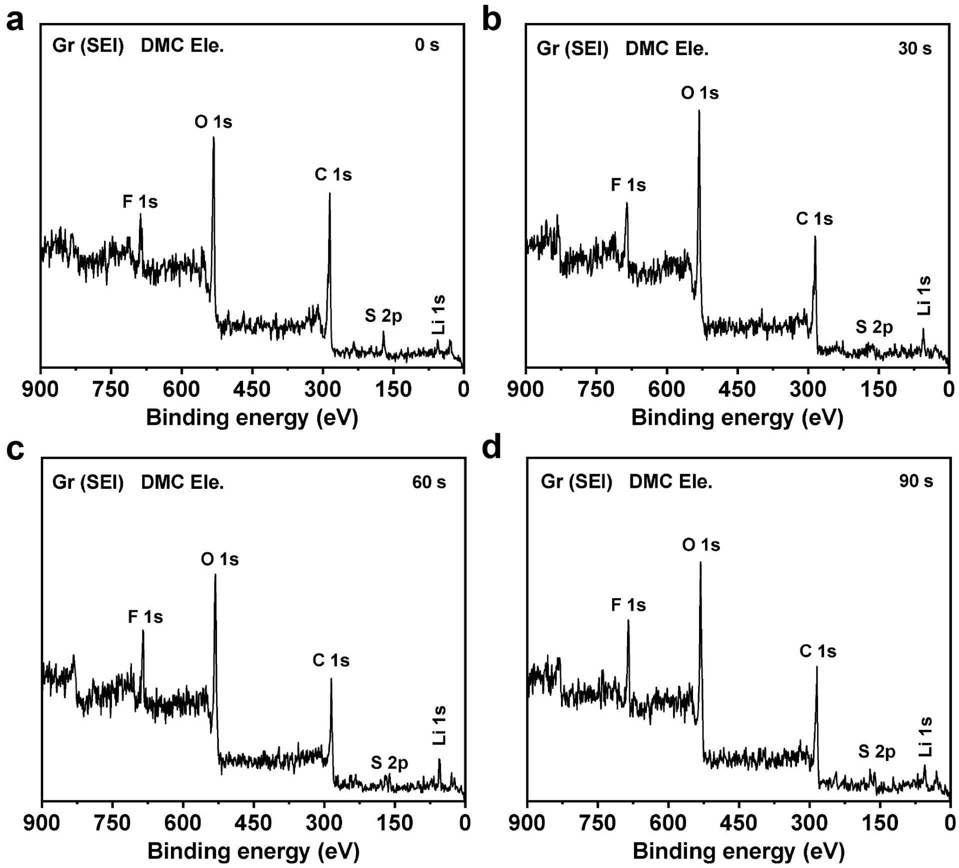

Figure S31. XPS full spectra of graphite anode cycled in DMC Ele..

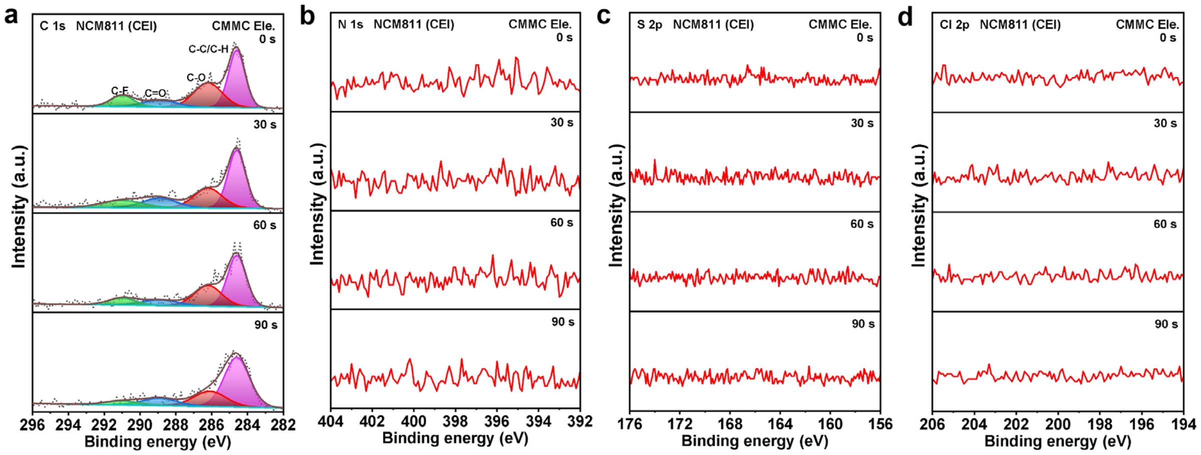

Figure S32. (a) C 1s, (b) N 1s, (c) S 2p, and d) Cl 2p XPS fine spectra of NCM811 cathode in CMMC Ele.. All elements have been calibrated according to the reference value (284.6 eV) for adsorbed carbon.

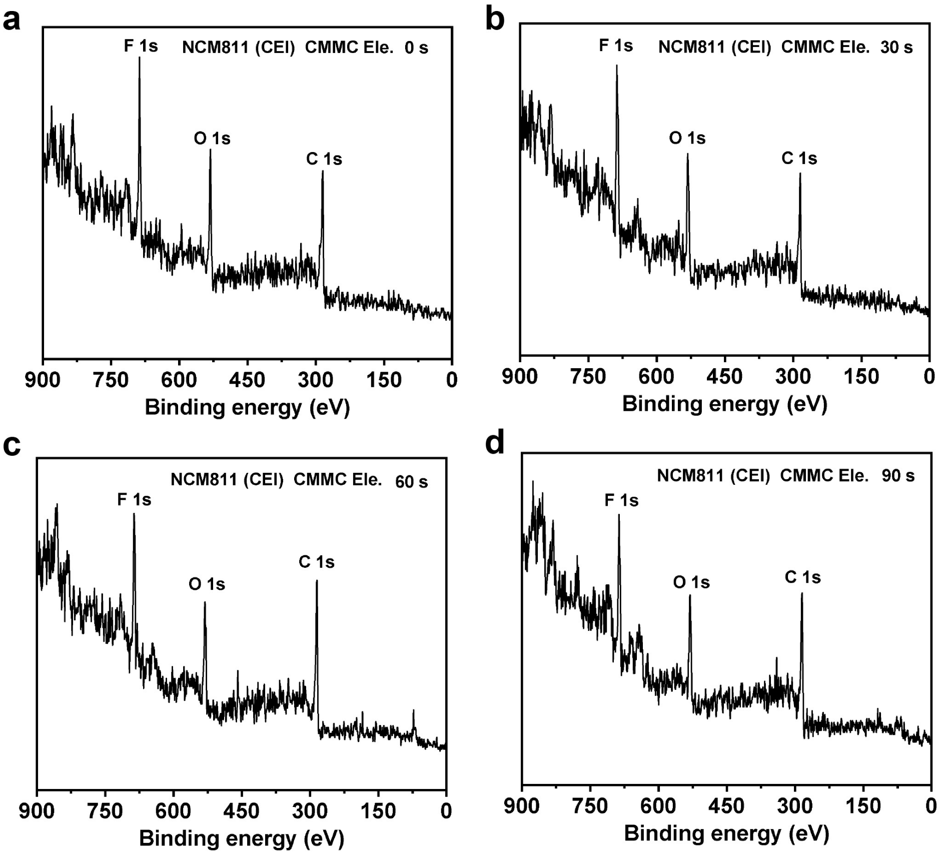

Figure S33. XPS full spectra of NCM811 cathode cycled in CMMC Ele..

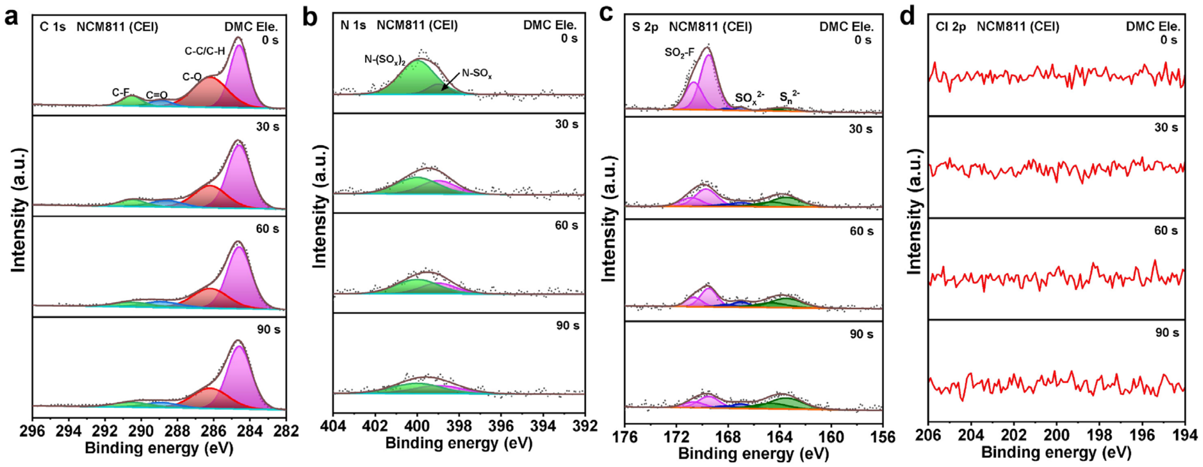

Figure S34. (a) C 1s, (b) N 1s, (c) S 2p, and d) Cl 2p XPS fine spectra of NCM811 cathode in DMC Ele.. All elements have been calibrated according to the reference value (284.6 eV) for adsorbed carbon.

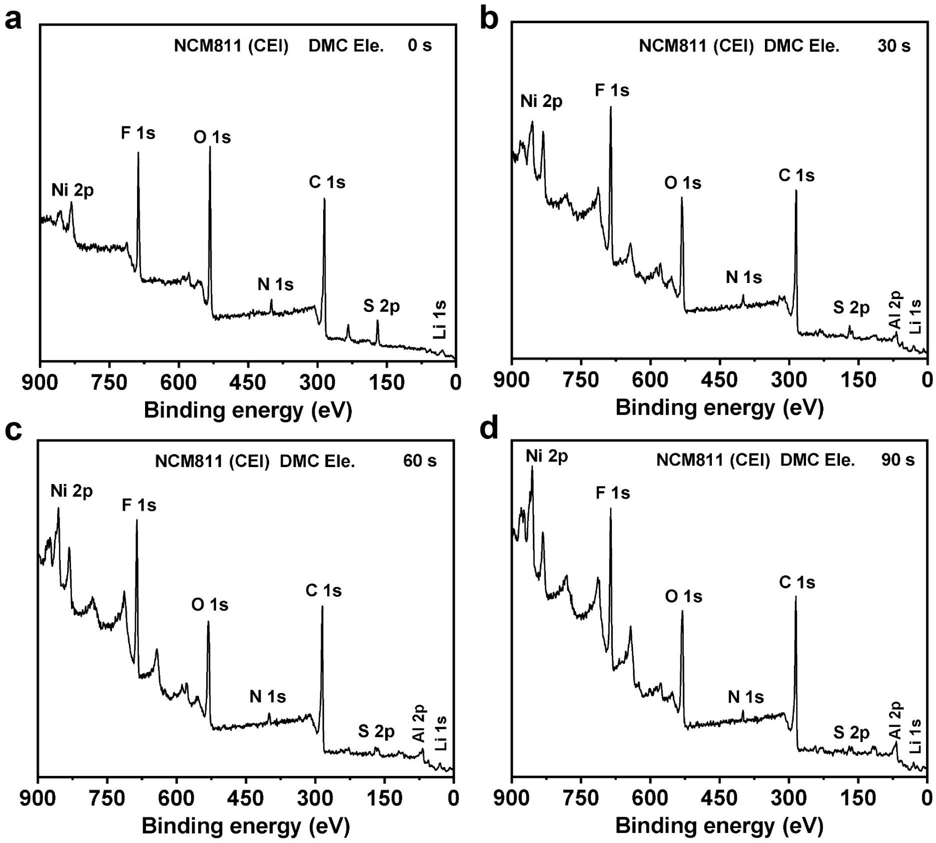

Figure S35. XPS full spectra of NCM811 cathode cycled in DMC Ele..

Table S6. Electrochemical performance comparison of flame-retardant electrolyte

Electrolyte formulation	Anode||Cathode	Cell type	Cutoff voltage	Capacity retention @Cycle number	References
$LiBF_4$ (2.04 M): TMS: FEC: TTFEPa (1:2:0.2:1 by mol.)	Gr||NMC811	CR2032	4.4 V	73.7%@500	[11]
1 M $LiPF_6$ + 3 vol% FEC in BMEC/EC (7 : 3 v/v)	Gr||NMC811	1 Ah-level pouch cell	4.2 V	91.4%@500	[12]
0.8 M $LiPF_6$ in EC/DEC/HCP (1:1.5:1 by weight)	Li||NMC811	CR2032	4.3 V	81.6%@150	[13]
1.44 M LiFSI in TMP_a-FEC-TTE (1.2:0.2:3.0 by mol.)	Gr||NMC811	CR2032	4.4 V	75.2%@500	[14]
1 M $LiPF_6$ /PC: FEMC: DFDEC (3:2:5 v/v/v)	Li||NMC811	CR2032	4.5 V	95%@100	[15]
1.2 M LiFSI/TEP_a-EC-BTFE (LiFSI-TEP_a 0.3EC-3BTFE, by mol.)	Gr||NMC811	CR2032	4.3 V	85.4%@300	[16]
LiFSI:TEP (1:2 by mol.) with 5% (by vol.) FEC and 0.05 M LiBOB	Gr||$LiCoO_2$	18650	4.2 V	90%@50	[17]
$LiPF_6$: LiDFOB: TMP: TTE = 0.7: 0.3: 4: 4 by mol.	Gr||NMC811	CR2032	4.3 V<br>4.6 V	80%@300<br>80%@200	[18]
1 M LiFSI in CMMC with 5 vol.% FEC (CMMC ele.)	Gr||NCM811	1.2Ah pouch cell	4.4 V	61.18%@800	This work

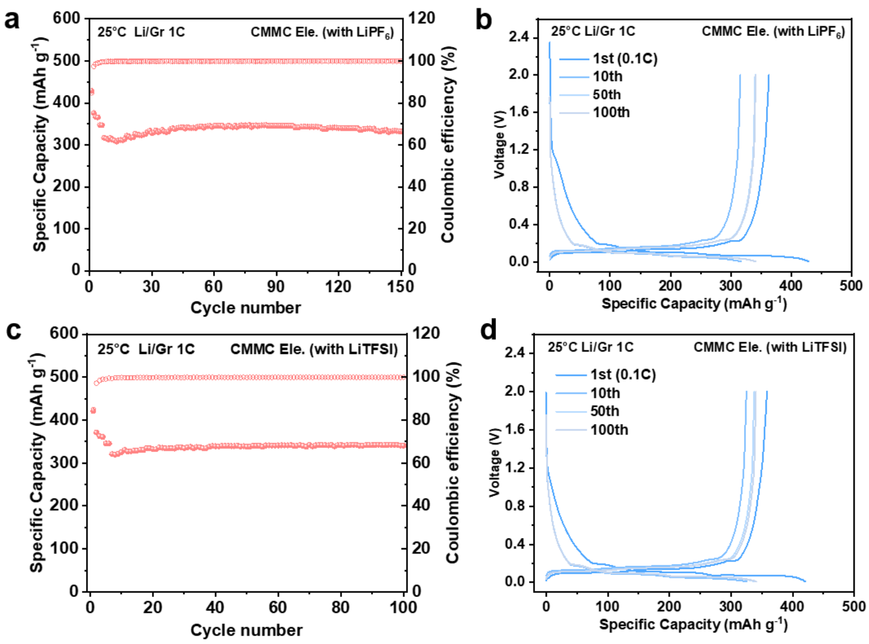

Figure S36. (a) Specific capacity and Coulombic efficiency of the Li/Gr half cell using CMMC Ele. (with $LiPF_6$) at 1 C. (b) The corresponding galvanostatic discharge/charge profiles with $LiPF_6$ at different cycles. (c) Specific capacity and Coulombic efficiency of the Li/Gr half cell using CMMC Ele. (with LiTFSI) at 1 C. (d) The corresponding galvanostatic discharge/charge profiles with LiTFSI at different cycles.

References

1. Zhao Y, Truhlar D. Density functionals with broad applicability in chemistry. *Acc Chem Res* 2008; **41**: 157−167.
2. Weigend F, Ahlrichs R. Balanced basis sets of split valence, triple zeta valence and quadruple zeta valence quality for H to Rn: design and assessment of accuracy. *Phys Chem Chem Phys* 2005; **7**: 3297−3305.
3. Lu T, Chen F. Multiwfn: A multifunctional wavefunction analyzer. *J Comput Chem* 2012; **33**: 580-592.
4. Berendsen H, Spoel D, Drunen R. GROMACS: A message-passing parallel molecular dynamics implementation. *Comput Phys Commun* 1995; **91**: 43-56.
5. Abraham M, Murtola T, Schulz R *et al*. GROMACS: high performance molecular simulations through multi-level parallelism from laptops to supercomputers. *SoftwareX* 2015; **1-2**: 19–25.
6. Wang J, Wolf R, Caldwell J *et al*. Development and testing of a general amber force field. *J Comput Chem* 2004; **25**: 1157–1174.
7. Wang J, Wang W, Kollman P *et al*. Automatic atom type and bond type perception in molecular mechanical calculations. *J Mol Graphics Modell* 2006; **25**: 247-260.
8. Singh U, Kollman P. An approach to computing electrostatic charges for molecules. *J Comput Chem* 1984; **5**: 129–145.
9. Besle B, Merz K, Kollman P. Atomic charges derived from semiempirical methods. *J Comput Chem* 1990; **11**: 431–439.
10. Humphrey W, Dalke A, Schulten K. VMD: visual molecular dynamics. *J Mol Graph* 1996; **14**: 33−38.
11. Qiu H, Jia H, Zhou Y *et al*. Exceptional battery-level safety of high energy density lithium-ion batteries through non-flammable and low-exothermic localize high concentration electrolytes. *Angew Chem Int Ed* 2025; **64**: e202423554.
12. Lee J, Jeon A-R, Lee H *et al*. Molecularly engineered linear organic carbonates as practically viable nonflammable electrolytes for safe Li-ion batteries. *Energy Environ Sci* 2023; **16**: 2924-2933.
13. Tan S, Tian Y, Zhao Y *et al*. Noncoordinating flame-retardant functional electrolyte solvents for rechargeable lithium-ion batteries. *J Am Chem Soc* 2022; **144**: 18240-18245.
14. Jia H, Xu Y, Zhang X *et al*. Advanced low-flammable electrolytes for stable operation of high-voltage lithium-ion batteries. *Angew Chem Int Ed* 2021; **60**: 12999-13006.

15. Pham H, Hwang E, Kwon Y *et al*. Approaching the maximum capacity of nickel-rich $LiNi_{0.8}Co_{0.1}Mn_{0.1}O_2$ cathodes by charging to high-voltage in a non-flammable electrolyte of propylene carbonate and fluorinated linear carbonates. *Chem Commun* 2019; **55**: 1256-1258.
16. Cao X, Xu Y, Zhang L *et al*. Nonflammable electrolytes for lithium ion batteries enabled by ultraconformal passivation interphases. *ACS Energy Lett* 2019; **4**: 2529-2534.
17. Zeng Z, Murugesan V, Han K *et al*. Non-flammable electrolytes with high salt-to-solvent ratios for Li-ion and Li-metal batteries. *Nat Energy* 2018; **3**: 674-681.
18. Cao S, Wen F, Ren X *et al.* Nonflammable dual-salt localized high-concentration electrolyte for graphite/$LiNi_{0.8}Co_{0.1}Mn_{0.1}O_2$ lithium-ion batteries: Li^+ solvation structure and interphase. J *Power Sources* 2023; **555**: 232392.
